# Inactivation of Cysteine Synthase CysK-A enhances flocculation, biofilm formation, and sensitivity to oxidative stress in *Azospirillum brasilense* Sp7

**DOI:** 10.1016/j.bioflm.2025.100335

**Published:** 2025-12-12

**Authors:** Job Herrera-Galindo, Clara Andrea Alcantara-Rosales, Oscar Mateo Ojeda, María Luisa Xiqui-Vázquez, Claudia Mancilla-Simbro, Sandra Reyes-Carmona, Beatriz Eugenia Baca, Alberto Ramírez-Mata

**Affiliations:** a Laboratorio de la Interacción Bacteria-planta, Centro de Investigaciones en Ciencias Microbiológicas, Instituto de Ciencias, Benemérita Universidad Autónoma de Puebla, Puebla, Mexico; b HybridLab. Fisiología y Biología Molecular de Células Excitables, Instituto de Fisiología. Benemérita Universidad Autónoma de Puebla., Puebla, Mexico

**Keywords:** *Azospirillum brasilense* Sp7, Cysteine metabolism, CysK-A protein, Biofilm formation, Exopolysaccharides, H_2_O_2_ stress, C-di-GMP

## Abstract

Cysteine biosynthesis is a critical metabolic pathway for bacterial physiology. However, the full impact on the lifestyle of the plant-beneficial bacterium *Azospirillum brasilense* Sp7 is not completely understood. Our previous work identified a cysteine synthase A (CysK-A) as a key enzyme in cysteine synthesis in *A. brasilense* Sp7, but its inactivation did not lead to cysteine auxotrophy, suggesting functional redundancy in this type of enzyme. Here, we comprehensively characterized an *A. brasilense* AR *cysK*-A mutant, revealing a multifaceted phenotype that highlights the indispensable role of CysK-A. The *cysK*-A mutant exhibited a growth defect that was rescued by genetic and chemical complementation, underscoring the importance of *de novo* cysteine synthesis for optimal metabolic fitness. Furthermore, the *cysK*-A mutant displayed a striking hyper-aggregative behavior, characterized by significantly enhanced flocculation, biofilm formation, and exopolysaccharide production. Confocal microscopy revealed an abundance of ovoid, cyst-like cells. This transition toward a sessile lifestyle, induced by cysteine limitation stress, correlated with the intracellular accumulation of cyclic-di-GMP, as determined by a c-di-GMP biosensor assay. Additionally, the *cysK*-A mutant exhibited increased sensitivity to exogenous hydrogen peroxide stress, a deficiency that was restored by *cysK*-A complementation. The mutation also led to enhanced adhesion to radish seeds; however, it did not result in statistically significant differences in overall radish seedling colonization after seven days, possibly due to compensatory mechanisms. Collectively, our findings establish CysK-A as crucial for optimal growth, stress tolerance, and the regulation of aggregative behaviors in *A. brasilense* Sp7, providing insights into the adaptive strategies employed by this important plant-associating bacterium.

## 1 Introduction

*Azospirillum brasilense* Sp7 is a model plant growth-promoting rhizobacterium (PGPR) widely used as a biofertilizer to improve crop productivity [1]. Its benefits are primarily attributed to its capacities for biological nitrogen fixation (BNF) and the synthesis of phytohormones, which collectively stimulate the development of the host's root system [2]. To persist in the competitive and fluctuating rhizosphere environment *A. brasilense* exhibits remarkable metabolic versatility and morphological plasticity, enabling its adaptation to various environmental stressors, such as desiccation, ultraviolet radiation, osmotic shock, and nutrient deficiency. These adaptations often involve the conversion into encapsulated cyst-like cells [1]. A key survival strategy is the transition from a planktonic state to a sessile lifestyle by forming biofilms. These structured communities, encased in a self-produced extracellular matrix, are critical for environmental persistence and efficient root colonization [3].

Cysteine, a sulfur-containing amino acid, is indispensable across nearly all domains of life. Beyond its fundamental incorporation into proteins, cysteine serves as a crucial precursor for a vast array of essential sulfur-containing compounds, including cofactors, secondary metabolites [4,5], and the antioxidant glutathione (GSH) [6]. In most bacteria, cysteine is synthesized *de novo* via a two-step pathway. First, serine acetyltransferase (CysE) converts l-serine into O-acetyl-l-serine (OAS). Subsequently, the pyridoxal 5′-phosphate (PLP)-dependent cysteine synthase (CysK) incorporates sulfide into OAS to produce l-cysteine [7]. Given its central metabolic position, disruption of cysteine biosynthesis can profoundly affect bacterial physiology, impacting growth, stress resistance, and cell-surface properties.

The importance of CysK extends beyond simple catalysis and is a conserved pathway across diverse bacteria. In pathogens such as *Brucella ovis* [8] and *Mycobacterium tuberculosis*, CysK isozymes are crucial for fitness during stationary phase, survival against oxidative stress, and maintaining cell wall integrity [9]. In the symbiotic bacterium *Vibrio fischeri*, CysK is directly implicated in biofilm formation, where a *cysK* mutant exhibits severe defects in wrinkled colony development. Notably, these defects are not always rescued by cysteine supplementation, suggesting a role for CysK beyond its catalytic function, potentially in regulating the production of biofilm matrix components [10].

Further complexity is seen in the regulation of this pathway. In *Providencia stuartii* and *Escherichia coli*, the CysE enzyme produces OAS, which can also act as an extracellular signal to regulate gene expression [11]. The existence of multiple CysK isozymes, such as CysK-A and CysK-B in *Salmonella typhimurium*, which are differentially expressed under aerobic and anaerobic conditions, highlights how bacteria fine-tune cysteine metabolism to adapt to fluctuating environments [12]. This functional diversity underscores which CysK can act as a metabolic hub integrating a nutritional status with complex adaptive behaviors.

While the multifaceted roles of cysteine metabolism are well-documented in other bacteria, this area remains largely unexplored in *Azospirillum*. Previous research in *A. brasilense* Sp7 identified a *cysK* gene encoding a cysteine synthase, named *cysK*-A. Unexpectedly, insertional inactivation of this gene did not lead to cysteine auxotrophy, raising questions about potential functional redundancy or the existence of alternative biosynthetic pathways [13].

Building upon this paradoxical finding, the present study aims to provide a comprehensive characterization of the physiological consequences of *cysK*-A inactivation in *A. brasilense* Sp7. We hypothesize that despite the lack of auxotrophy, CysK-A plays a critical, pleiotropic role in the bacterium's lifestyle. To test this, we investigated the impact of the *cysK*-A mutation on growth kinetics, cellular aggregation (flocculation), biofilm formation, EPS production, tolerance to oxidative stress, plant-microbe interactions, including adhesion and root colonization. We also explored potential regulatory mechanisms underlying the observed phenotypes, specifically by examining intracellular c-di-GMP levels and performing a bioinformatic search for additional putative *cysK* genes that might contribute to cysteine homeostasis. This research seeks to unravel the intricate connection between a core metabolic enzyme and the complex adaptive traits that underpin the success of *A. brasilense* as a PGPR.

## 2 Materials and Methods

### 2.1 Bacterial strains and growth conditions

Strains and plasmids used in this work are described in Table 1. *Escherichia coli* strains were propagated at 37 °C in Luria-Bertani (LB) broth. *Azospirillum brasilense* Sp7 and derived strains (*A. brasilense* AR *cysK*-A mutant, *A. brasilense* AR pAB-*cysK* complemented) were routinely grown in Congo red (CR) medium, MD medium, or minimal K-malate medium [[14], [15], [16]]. When required, antibiotics and other compounds were added to the growth media at the following final concentrations: Kanamycin (Km) 50 μg/mL; Tetracycline (Tc), 15 μg/mL; Gentamycin (Gm) 30 μg/mL, Calcofluor (CF) 200 μg/mL, Cysteine 0.5 mM.

**Table 1:** Strains and plasmids.

Strain or Plasmid	Description	Antibiotic resistance	Reference or source
*Escherichia coli* S17.1	*E. coli* Res_ Mod1 *recA* proA thi, Sm, Sp, Tp integrated plasmid RP4 Tc:Mu Km:Tn7 Tra1		[17]
*Azospirillum brasilense* Sp7	Wild-type strain ATCC29145		[18]
*A. brasilense* AR	Derivative strain *cysK*::*km* of *A. brasilense* Sp7	Km	[13]
*A. brasilense* AR (pAB-*cysK*)	Derivative strain from *A. brasilense* AR harboring the pAB-*cysK* plasmid	Km, Tc	[13]
*A. brasilense* AR pVK-EV	Derivative strain from *A. brasilense* AR harboring the empty pVK100 vector	Km, Tc	[13]
*A. brasilense* Sp7 pMP2449-5	Derivative strain from *A. brasilense* Sp7 harboring the pMP2449-5 plasmid, which contains the *mCherry* gene	Gm	This study
*A. brasilense* AR pMP2449-5	Derivative strain from *A. brasilense* AR harboring the pMP2449-5 plasmid, which contains the *mCherry* gene	Km, Gm	This study
*A. brasilense* Sp7 pFY4535	Derivative strain from *A. brasilense* Sp7 harboring the pFY4535 plasmid, which contains the c-di-GMP biosensor	Gm	This study
*A. brasilense* AR pFY4535	Derivative strain from *A. brasilense* AR harboring the pFY4535 plasmid, which contains the c-di-GMP biosensor	Km, Gm	This study
pAB-*cysK*	Vector containing the *cysK* gene from a genomic library of *A. brasilense* Sp7	Tc	[13]
pMP2449-5	Plasmid broad host range, containing *mCherry* gene expressed under control of lac promoter, cloned in pBBR-1	Gm	[19]
pFY4535	Plasmid broad host range, containing the c-di-GMP biosensor	Gm	[20]

Kanamycin (Km); Tetracycline (Tc); Gentamycin (Gm).

### 2.2 Growth curves

Growth of wild-type (*A. brasilense* Sp7), mutant (*A. brasilense* AR), genetically complemented (*A. brasilense* AR-pAB-*cysK*), control with empty vector (*A. brasilense* AR-EV), and chemically complemented (*A. brasilense* AR + Cysteine 0.5 mM). Bacterial cells from 18-h-old MD medium were inoculated into 100 mL Erlenmeyer flasks containing 25 mL of K-malate or K-lactate liquid media. The initial optical density at 600 nm (OD_600_) was adjusted to 0.05. Cultures were incubated at 30 °C in a rotary shaker at 150 rpm, and OD_600_ measurements were taken every 2–3 h. Each experiment was conducted at least three times independently, with two technical replicates per experiment. Representative growth curves are shown for each set of strains.

### 2.3 Colony-forming unit (CFU) count

Bacterial strains (*A. brasilense* Sp7, *A. brasilense* AR, genetically complemented *A. brasilense* AR-pAB-*cysK*, and chemically complemented *A. brasilense* AR + Cysteine 0.5 mM) were initially grown for 18 h in MD medium. These starter cultures were used to inoculate 100 mL Erlenmeyer flasks containing 25 mL of K-lactate liquid medium. The flasks were incubated for 24 h at 30 °C with (150 rpm). For CFU enumeration, the 24-h cultures were serially diluted in K-lactate medium. This was initiated by adding 100 μL of culture to 900 μL of medium (a 10^−1^ dilution) and continued in steps up to a 10^−13^ dilution. From the appropriate serial dilutions, five 10 μL aliquots were spot-plated onto Congo red agar plates. Following incubation, colonies within each spot were counted. These counts were used to calculate the concentration of viable cells, expressed as CFU/mL, for each assessed strain.

### 2.4 Biofilm assays

Biofilm formation assays were conducted as described previously [21]. Briefly, bacteria were cultured overnight in LB∗ medium (Luria-Bertani broth supplemented with 10 mM, CaCl_2_, and 10 mM, MgCl_2_). Subsequently, 7.5 mL of Nfb∗ medium (supplemented with KNO_3_ or NH_4_Cl) in borosilicate round-bottom tubes were inoculated with 75 μL of overnight cultures diluted to an OD_600_ of 2.0. The inoculated cultures were incubated for 5 days at 30 °C under static conditions. Biofilms were stained with a 0.05 % (w/v) crystal violet solution for 30 min and then rinsed with sterile distilled water. Bound crystal violet was solubilized with 2 ml of 33 % (v/v) acetic acid, and its concentration was determined using a 96-well microplate reader (EON, BioTek, Winooski, VT, USA) at 595 nm. Crystal violet measurements were normalized to total protein concentration as determined by the Bradford method [22]. The results presented are based on three independent experiments with three biological replicates each.

### 2.5 Biofilm formation and exopolysaccharide analysis by CLSM

*Azospirillum* biofilms were cultivated on FluoroDishes (Thermo Fisher Scientific) under conditions specified in the biofilm assay section [23]. To visualize biofilm formation and exopolysaccharide production, cultures were stained with Calcofluor to a final concentration of 85 μM, and 1 mM of propidium iodide (PI) (Sigma Aldrich, United States). After five days of static incubation at 30 °C, three-dimensional biofilm structures were observed using an Eclipse Ti-E C2+ confocal laser scanning microscope (Nikon) equipped with CFI Plan Apo VC 20x/1.2 and CFI Plan Apo VC 60x/1.2 WI objective lenses. Excitation and emission wavelengths were 380/475 nm for Calcofluor and 535/617 nm for PI. During imaging, the temperature of the biofilm culture dish was maintained at 30 °C using a large cage incubator and a stage-top incubator. Z-stacks were acquired at 0.5 μm intervals. Three-dimensional structures were reconstructed using NIS Elements imaging software for Nikon microscopy (Nikon, Japan). For comparative analysis of treatments, maximum projection images were rendered from the 3D datasets.

### 2.6 Calcofluor binding assay

To assess exopolysaccharides production, *Azospirillum* strains were pre-cultured in MD liquid medium at 30 °C for approximately 18–20 h. Bacteria were then harvested, resuspended in phosphate-buffered saline (PBS), adjusted to an OD_600_ of 2.0, and subsequently diluted 1:100. Ten microliters of this cell suspension were spotted onto solid LB medium lacking NaCl, supplemented with calcofluor (CF; 200 μg/ml) [16]. Calcofluor binding was observed under ultraviolet (UV) light. CF plates were photographed after 5 days of incubation at 30 °C.

### 2.7 Biofilm viability assessment by live/dead staining

*Azospirillum* biofilms were cultured on fluorodishes (Thermo Fisher Scientific) for five days under static conditions at 30 °C, as specified in the biofilm assay section (23). To assess cell viability and differentiate between living and non-living cells, the biofilms were stained with two nucleic acid dyes. Propidium iodide (PI, 1 mM, Sigma Aldrich), which penetrates membrane-compromised (non-living) cells, was used in combination with 4′, 6-diamidino-2-phenylindole dihydrochloride dye (DAPI, Calbiochem), which serves as a counterstain for all bacterial cells. The three-dimensional biofilm structures were observed using an Eclipse Ti-E C2+ confocal laser scanning microscope (Nikon) equipped with CFI Plan Apo VC 20x/1.2 and CFI Plan Apo VC 60x/1,2 WI objective lenses. Fluorescence was detected using excitation/emission wavelengths of 359/461 nm for DAPI and 535/617 nm for PI. During imaging, the temperature of the biofilm culture dish was maintained at 30 °C using a large cage incubator and a stage-top incubator. Z-stacks were acquired at 0.5 μm intervals. Three-dimensional structures were reconstructed using NIS Elements imaging software for Nikon microscopy (Nikon, Japan).

### 2.8 H_2_O_2_ susceptibility test (disk diffusion assay)

*Azospirillum* strains were grown overnight in MD liquid medium at 30 °C. Bacteria were harvested, resuspended in PBS, and adjusted to an OD_600_ of 2.0. A 100 μL aliquot was spread evenly over the surface of K-malate agar plates. A 10 μL of 1 % H_2_O_2_ was spotted onto a sterile paper disk, and two disks were placed on the plate. The diameter of growth inhibition zone around disk was measured after a 24-h incubation at 30 °C.

### 2.9 Determination of cyclic di-GMP levels using a c-di-GMP biosensor

To generate *A. brasilense* strains harboring the c-di-GMP biosensor plasmid pFY4535 [20] conjugation was performed between the donor strain of *E. coli* S17.1 (pFY4535) and the recipient strains *A. brasilense* Sp7 and *A. brasilense* AR, as previously described [16]. Following conjugation, reporter strains were isolated by streaking onto K-lactate agar plates supplemented with 30 μg/mL gentamicin and incubated at 30 °C for 72 h. Two representative colonies from each plate were used to inoculate pre-cultures in MD liquid medium, which were incubated at 30 °C for 18–20 h. Bacterial cells were then harvested, resuspended in PBS, and adjusted to an OD_600_ of 2.0. This suspension was subsequently diluted 1:100. Ten microliters of this suspension were spotted onto solid Nfb∗+KNO_3_ medium containing 30 μg/mL gentamicin. The plates were incubated at 30 °C, and colony growth was monitored for 5 days. The intensity of the red bacterial colony stain, resulting from TurboRFP expression, directly correlates with intracellular c-di-GMP concentrations as detected by the biosensor [20,24]. For microscopic analysis, biosensor fluorescence was assessed using a Nikon Eclipse TE2000U fluorescence microscope. A portion of a colony from each strain was resuspended in 15 μL of sterile distilled water. This suspension was deposited on a coverslip and covered with a 1 % agarose plug. Fluorescence was recorded using two channels: the calibrator AmCyan fluorophore (Excitation: 457 nm; Emission: 520 nm) and the reporter TurboRFP fluorophore (Excitation: 553 nm; Emission: 574 nm). Obtained images were processed and analyzed using Nikon NIS Elements software.

### 2.10 Adherence analysis of *A. brasilense* strains to radish seeds

To assess the adherence of *Azospirillum* strains to radish seeds (*Raphanus sativus* L.), the following protocol was employed. *Azospirillum* strains were cultured in MD medium until the early stationary phase of growth was reached, to an OD_600_ of 1.0. These cultures were then diluted in K-malate medium to achieve a final cell density of 5 X 10^7^ colony-forming units per milliliter (CFU/mL). Prior to the adherence assay, radish seeds were surface sterilized to eliminate contaminating microorganisms. Briefly, 50 seeds were treated with 50 mL of a 2 % (v/v) liquid detergent solution (Extran MA-02, Merck) in sterile deionized water for 2 min, followed by three washes with sterile deionized water. Subsequently, the seeds were incubated in a 1 % (v/v) sodium hypochlorite solution with agitation at 60 rpm for 5 min. Following, the seeds were rinsed eight times with sterile demineralized water (5 min per rinse). Ten surface-sterilized seeds per *Azospirillum* strain were immersed in 4 mL of K-malate medium containing approximately 10^7^ CFU/mL of the respective strain and incubated at room temperature with shaking at 60 rpm for 2 h. After the adherence period, the seeds were carefully washed once with a PBS solution (pH 6.8). The adhered bacteria were quantified by colony counting on Congo Red (CR) medium supplemented with the corresponding selective antibiotic: ampicillin (50 μg/mL) for *A. brasilense* Sp7, kanamycin (50 μg/mL) for *A. brasilense* AR, and tetracycline (15 μg/mL) for the *A. brasilense* AR pAB-*cysK* complemented strain [19].

### 2.11 Plant colonization experiments under soil conditions

To determine the colonization of radish seedlings by *A. brasilense* strains, the seeds were prepared as described above and then placed on Petri plates containing 0.6 % (w/v) water-agar. These plates were incubated in the dark for two days to allow for seed germination. Subsequently, germinated seedlings were carefully transferred to sterile soil obtained from the Campo Agrícola Experimental del Valle de México (INIFAP). To remove any loosely adhering soil particles, the plants were gently washed with sterile water for 40 s. The colonizing bacteria were then quantified by colony counting on CR medium supplemented with the appropriate antibiotic, as described [24].

### 2.12 Colonization of *A. brasilense* strains on radish seedlings analysis by CLSM

The colonization assays of *Azospirillum* on radish seedlings were conducted essentially as previously described [19], using radish seeds in a hydroponic system. Briefly, the seeds underwent surface sterilization, were placed on Petri dishes filled with water-agar 0.6 % (w/v) and incubated in darkness for two days. Germinated seedlings were transferred into glass tubes (20 × 150 mm) containing 15 mL of Hoagland hydroponic solution supplemented with 25 mM KNO_3_ and 0.5 % agar, following the protocol outlined by Arsène et al. (1994) [25]. The tubes were covered by additional test tubes that were larger in diameter (30 × 200 mm). For seven days, the seedlings were maintained in a controlled growth chamber at 28 °C under a 12-h photoperiod with 70 % relative humidity. The plant roots (nine plants per strain across three independent biological replicates) were embedded with approximately 10^7^ CFU/mL of *A. brasilense* Sp7 (pMP2449-5) and *A. brasilense* AR (pMP2449-5). Subsequently, the colonizing bacteria were evaluated through microscopy analysis. Root samples were excised, rinsed in sterile H_2_O, mounted on glass slides with PBS (pH 6.8), and covered with glass coverslips. Confocal images were acquired using a Nikon C2+ CLSM (Nikon, Tokyo, Japan) equipped with a CFI Plan Apo Lambda 20 × objective and two helium–neon lasers for the excitation of the mCherry fluorophore at wavelengths of 540 nm and 650 nm and an argon laser for the excitation of radish autofluorescence at 488 nm. The cell morphology and localization of the *A. brasilense* strains were evaluated in the roots to assess the presence of the bacteria and biofilms [23,24].

### 2.13 Bioinformatic analysis of CysK and CysE homologs

To identify putative CysK and CysE homologs in the *A. brasilense* Sp7 genome [26], a BLASTP search was conducted against the NCBI non-redundant protein database. The identified protein sequences (listed in Table S1 and Table S3) were subjected to a multiple sequence alignment using Clustal Omega to evaluate their conservation [27]. Subsequently, we conducted genomic organization analyses of the *cysE* and *cysK* genes in *A. brasilense* Sp7 genome. Additionally, three-dimensional (3D) structural models of the putative CysK proteins were generated using AlphaFold-3 [28]. These predicted models were then analyzed and validated using Foldscript v1.2 [29]. For comparative structural analysis and figure generation, the AlphaFold-3 models were superimposed onto relevant crystallographic structures obtained from the Protein Data Bank (PDB). All structural visualization, analysis, and image rendering were performed using UCSF Chimera X v1.8 [30].

### 2.14 Statistical analysis

To identify statistically significant differences between the examined strains, the data were analyzed with a Student's t-test. Differences were deemed significant at a threshold of *p* < 0.05. The analysis was conducted using SigmaPlot software (version 14.5).

## 3 Results

### 3.1 Inactivation of *cysK*-A gene impairs growth and reduces cell viability

**Fig. 1:**
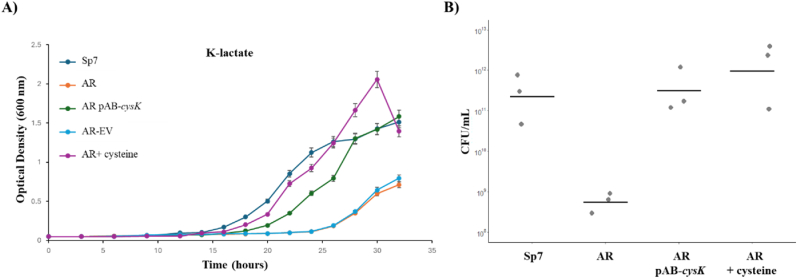
**Inactivation of *cysK*-A impairs the growth of *A. brasilense*, a defect rescued by genetic complementation and the addition of cysteine to the growth medium.** (**A**) Growth curves in liquid minimal medium containing lactate as the carbon source. In the graphs, strains are represented as follows: wild-type *A. brasilense* Sp7 (dark blue), *A. brasilense* AR (orange), *A. brasilense* AR-pAB-*cysK* (green), *A. brasilense* AR with 0.5 mM cysteine (purple), and *A. brasilense* AR-EV (light blue). Error bars represent the standard deviation of technical replicates from a representative experiment. (**B**) Colony-forming units (CFU) were counted after 24 h of incubation at 30 °C and 150 rpm in K-lactate medium. The strains used were *A. brasilense* Sp7, *A. brasilense* AR, *A. brasilense* AR-pAB-*cysK*, and *A. brasilense* AR supplemented with 0.5 mM cysteine. (For interpretation of the references to color in this figure legend, the reader is referred to the Web version of this article.)

To assess the physiological role of *cysK*-A in *A. brasilense*, we monitored the growth kinetics of the wild-type strain (*A. brasilense* Sp7), the *cysK*-A mutant (*A. brasilense* AR), a genetically complemented strain (*A. brasilense* AR pAB-*cysK*)*,* a chemically complemented (*A. brasilense* AR + 0.5 mM Cysteine), and an empty vector control (*A. brasilense* AR pVK-EV). The strains were cultured in minimal medium using either K-lactate (Fig. 1A) or K-malate (Fig. S1) as the sole carbon source. In both conditions, the *cysK*-A mutant exhibited a pronounced growth defect compared to the wild-type strain. Specifically, the mutant reached a significantly lower final optical density, indicating reduced culture yield (Fig. 1A and S1). This growth impairment was fully restored to wild-type levels in the genetically complemented strain, which expresses the *cysK*-A gene in trans. In contrast, the empty vector control behaved identically to the *cysK*-A mutant (Fig. 1A, and S1). To confirm that the reduced optical density correlated with a decrease in viable cells, we quantified CFU/mL after 24 h of growth in K-lactate minimal medium. The *cysK*-A mutant (6.1 × 10^8^ CFU/mL) showed a significant, ∼ 3-log reduction in cell viability compared to the wild-type strain (3.9 × 10^11^ CFU/mL). This defect was successfully rescued in both the genetically complemented strain (5.2 × 10^11^ CFU/mL) and the chemically complemented strain (via the addition of 0.5 mM cysteine; 2.03 × 10^12^ CFU/mL) (Fig. 1B). These results demonstrate that a functional CysK-A, and a complete *de novo* cysteine biosynthesis pathway are essential for optimal planktonic and metabolic fitness in *A. brasilense* Sp7.

### 3.2 *A. brasilense* AR exhibits increased flocculation and biofilm formation under free-living conditions

**Fig. 2:**
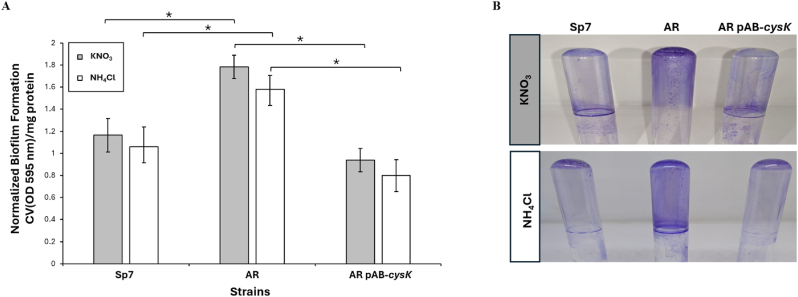
Impact of *cysK* mutation on biofilm formation. Biofilm production by the wild-type (*A. brasilense* Sp7), *A. brasilense* AR mutant, and *A. brasilense* AR-pAB-*cysK* complemented strains were evaluated in static cultures. (**A**) Quantification of biofilm formation using a crystal violet (CV) staining assay. Strains were cultured for 5 days at 30 °C in Nfb∗ minimal medium supplemented with either KNO_3_ (gray bars) or NH_4_Cl (white bars) as the nitrogen source. Adherent biomass was stained with 0.05 % (w/v) CV, and the absorbance was measured at 540 nm. The data was normalized with total protein determined by Bradford assay. Error bars represent the standard deviation from three independent experiments. Asterisks indicate a statistically significant difference compared to the wild-type strain under the same conditions (*p* < 0.05, Student's t-test). (**B**) Representative images of CV-stained biofilms formed in glass tubes, corresponding to the conditions quantified in panel A. (For interpretation of the references to color in this figure legend, the reader is referred to the Web version of this article.)

During routine cultivation in minimal media, the *A. brasilense* AR mutant strain was observed to form flocs compared to the wild-type strain. This observation prompted an investigation into whether the mutant strain exhibited enhanced flocculation and biofilm formation. Consequently, the flocculation and biofilm-forming capabilities of the mutant, wild-type, and complemented strains were assessed. The *A. brasilense* AR mutant strain demonstrated significantly greater flocculation than the wild-type strain. This phenotype was reverted to wild-type levels in the complemented strain, indicating a *cysK*-A dependent effect (Fig. S2). Similarly, the mutant strain formed considerable biofilm mass compared to the wild-type strain in media supplemented with two different types of nitrogen sources (KNO_3_ or NH_4_Cl). Complementation with the wild-type *cysK*-A gene also restored this phenotype to wild-type levels (Fig. 2). These data strongly suggest that the absence of the *cysK*-A gene enhances both flocculation and biofilm formation. This phenomenon may be attributed to an impaired *de novo* cysteine synthesis capacity in the mutant, a deficiency that is rectified by genetic complementation with the wild-type *cysK*-A gene.

### 3.3 A *cysK*-A mutation enhances exopolysaccharide production in *A. brasilense*

**Fig. 3:**
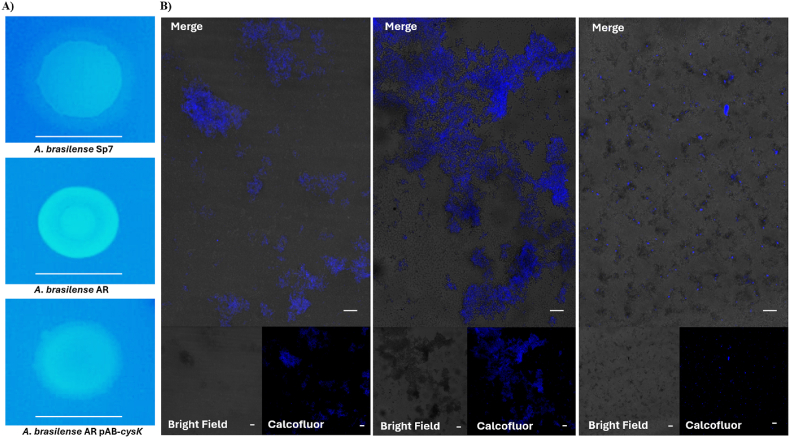
Colony and biofilm calcofluor staining assays. Production of exopolysaccharide (EPS) by the wild-type (*A. brasilense* Sp7), the *cysK-A* mutant (*A. brasilense* AR), and the complemented (*A*. *brasilense* AR-pAB-*cysK*) strains were visualized by staining with calcofluor, which binds to β-linked polysaccharides. (**A**) Colonies were grown for 5 days at 30 °C on solid LB medium (lacking NaCl) supplemented with Calcofluor (200 μg/mL). Images were captured under UV illumination to visualize fluorescence. Scale bar = 10 mm. (**B**) Confocal micrographs of biofilms grown for 5 days at 30 °C on agar-solidified Nfb∗ medium containing Calcofluor (85 μM). The biofilms were observed with a Nikon Eclipse Ti-E C2+ microscope. Scale bar = 20 μm.

Exopolysaccharides (EPS) on the cell surface are known to be critical for key developmental processes in *A. brasilense* Sp7, including aggregation, flocculation, biofilm, and the formation of cyst-like cells [23,[31], [32], [33], [34], [35]]. To investigate the molecular basis for increased biofilm formation in a *cysK*-A mutant, we evaluated its production of specific EPS using Calcofluor dye. When cultured on Nfb∗ medium with KNO_3_, the *cysK*-A mutant colonies displayed significantly increased binding of Calcofluor relative to the wild-type strain. This phenotype was restored to wild-type levels in the complemented strain (Fig. 3). As Calcofluor specifically binds to polysaccharides with β-1,3 and β-1,4 linked glycans [36,37], these results strongly indicate that the *cysK* mutation leads to the overproduction of such an exopolysaccharide. Future work will focus on the isolation and structural characterization of this polymer.

### 3.4 *cysK*-A mutant promotes an increase in biofilm thickness

To investigate the role of the *cysK*-A gene in biofilm development, we compared the biofilm architecture of the *A. brasilense* AR mutant with its wild-type and complemented counterparts. Biofilms were cultivated in Nfb∗ medium with KNO_3_ and analyzed by confocal microscopy. Staining with Calcofluor revealed that the *cysK*-A mutant produced a hyper-aggregative phenotype with significantly increased fluorescence compared to the wild-type strain (Fig. 3B). This suggests that the loss of the *cysK*-A gene induces a stress response that elevates exopolysaccharide (EPS) production. Consistent with this observation, quantitative analysis demonstrated a 22 % increase in biofilm thickness in the mutant. Both the hyper-aggregative phenotype and the increased thickness were reverted to wild-type levels in the complemented strain, confirming that these effects are directly attributable to the mutation of the *cysK*-A gene (Fig. S3).

**Fig. 4:**
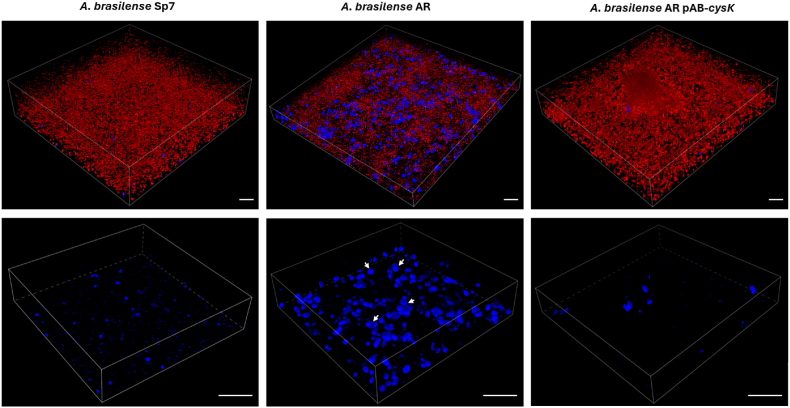
Three-dimensional (3D) reconstruction of *A. brasilense* biofilms by CLSM. Biofilms were stained to visualize some matrix components: red fluorescence indicates extracellular DNA (eDNA) stained with propidium iodide, and blue fluorescence indicates exopolysaccharides stained with Calcofluor. Upper panels: composite 3D images captured at 20x magnification, showing the distribution of both eDNA and EPS. Lower panels: Detailed 3D images of exopolysaccharides captured at 60x magnification. White arrows indicate structures resembling cyst-like cells within the biofilm of the *cysK*-A mutant strain. Scale bar = 25 μm. (For interpretation of the references to color in this figure legend, the reader is referred to the Web version of this article.)

We further explored *Azospirillum* cellular morphology within the biofilm by co-staining with propidium iodide (PI) to label dead or membrane-compromised cells. The PI staining was augmented in the *cysK*-A mutant compared with the wild-type and genetically complemented strains, indicating reduced cell viability. Furthermore, the *cysK*-A mutant displayed a notable abundance of ovoid-shaped cells. These probable cyst-like structures co-localized with intense calcofluor fluorescence, suggesting they are encased in a thick EPS layer (Fig. 4, S4, and Videos 1 and 2).

Supplementary data related to this article can be found online at https://doi.org/10.1016/j.bioflm.2025.100335

The following are the Supplementary data related to this article:

Multimedia component 2 Multimedia component 2

Multimedia component 3 Multimedia component 3

Taken together, our results demonstrate that the absence of CysK-A triggers a significant shift in biofilm development, characterized by enhanced EPS production and increased structural thickness. This is likely a protective strategy against cysteine starvation.

### 3.5 *cysK*-A inactivation reduces bacterial cell viability in *A. brasilense* biofilm

To confirm the presence of reduced cell viability in the *cysK*-A mutant biofilm, we performed a bacterial viability assay using Propidium Iodide and DAPI co-staining, visualized by confocal microscopy. In this assay, the *cysK*-A mutant biofilm showed a marked increase in red fluorescence intensity corresponding to the PI fluorophore, which indicates a higher proportion of non-viable bacteria. In contrast, the wild-type (*A. brasilense* Sp7) strain's biofilm exhibited a lower proportion of non-viable bacteria and a higher number of viable cells, as denoted by DAPI staining. This effect was restored in the genetically complemented strain (*A. brasilense* AR-pAB-*cysK*), which showed a lower proportion of non-viable cells (Fig. S4). These data confirm that an intact *cysK*-A gene is necessary to maintain cellular homeostasis and metabolic fitness during *A. brasilense* biofilm formation. Furthermore, these results suggest that the increased biofilm formation observed in the *cysK*-A mutant may be associated with increased release of extracellular DNA (eDNA), which could contribute to the composition of the extracellular matrix [15]. However, this hypothesis requires confirmation in future studies.

### 3.6 Loss of the *cysK*-A gene promotes an increase in cellular c-di-GMP levels in *A. brasilense*

**Fig. 5:**
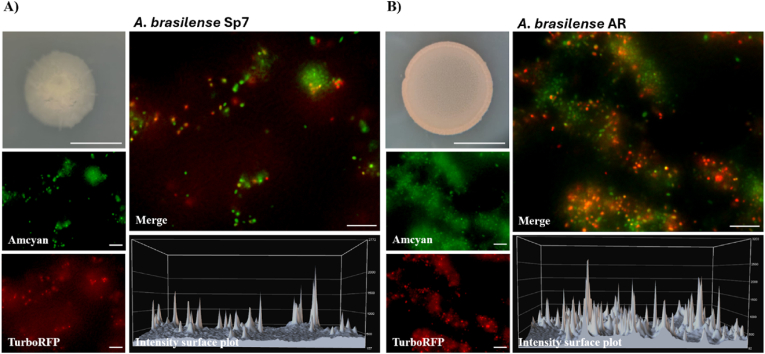
***A. brasilense* AR mutant exhibits elevated levels of intracellular c-di-GMP.** Colony morphology of wild-type *A. brasilense* Sp7 (**A**) and the AR mutant (**B**) expressing a c-di-GMP biosensor (pFY4535). Cultures were grown for five days at 30 °C on Nfb∗ agar supplemented with KNO_3_. The intracellular c-di-GMP concentration is proportional to the expression of TurboRFP (red), resulting in a red colony color. The AR mutant's more intense coloration indicates higher c-di-GMP accumulation than in the WT. Representative fluorescence microscopy of individual WT and AR mutant cells. Images were captured on a Nikon Eclipse TE2000-U microscope using the following excitation/emission wavelengths: 489/519 nm for AmCyan (green) and 553/574 nm for TurboRFP (red). Gray images are intensity surface plots corresponding to the fluorescence emitted by TurboRFP. Images were processed using Nikon NIS Elements software. All images shown are representative of three independent experiments. Scale bars, 10 mm (colonies); 10 μm (individual cells). (For interpretation of the references to color in this figure legend, the reader is referred to the Web version of this article.)

A mutation in the *cysK*-A gene in *A. brasilense* leads to increased biofilm formation and exopolysaccharide production. This observation prompted us to investigate the intracellular levels of the second messenger c-di-GMP using a specific biosensor, given that elevated c-di-GMP is known to significantly enhance these phenotypes. To determine cellular c-di-GMP accumulation, we introduced the c-di-GMP biosensor plasmid pFY4535 into the wild-type *A. brasilense* Sp7 and the *cysK*-A mutant *A. brasilense* AR strains, as detailed in Materials and Methods. Both strains were cultured for 5 days on solid Nfb∗ medium supplemented with KNO_3_. Subsequent macroscopic and microscopic analyses revealed that the *cysK*-A mutant developed a distinct red pigmentation. This coloration, which was reduced in the WT strain, indicates enhanced TurboRFP protein production from the biosensor and thus suggests elevated intracellular c-di-GMP levels in the mutant (Fig. 5A and B). We suggest that this c-di-GMP accumulation mediates the observed increase in biofilm formation and EPS synthesis. This effect is likely a consequence of *cysK*-A loss and the probable concomitant decrease in *de novo* cysteine biosynthesis.

### 3.7 *A. brasilense* AR mutant strain exhibits increased sensitivity to exogenous H_2_O_2_ stress

Cysteine is a crucial amino acid, not only for protein synthesis but also as one of the three amino acids that comprise glutathione. Glutathione plays a central role in mitigating various bacterial stressors, including oxidative stress. We hypothesized that defects in cysteine biosynthesis in *Azospirillum* would impair glutathione synthesis, thereby increasing cellular susceptibility to oxidative stress. To assess whether the *cysK*-A mutant was more sensitive to H_2_O_2_ stress compared to the wild-type, we employed a disc diffusion assay (Fig. S5A). The wild-type strain exhibited an inhibition zone diameter of (2.8 ± 0.15) cm, while the *A. brasilense* AR mutant displayed a significantly larger zone of (3.8 ± 0.1) cm, which was significantly different based on a Student's t-test (*p <* 0.01) (Fig. S5B). This heightened sensitivity was restored to wild-type levels upon complementation with the *cysK*-A gene, indicating that CysK-A contributes to H_2_O_2_ tolerance in *A. brasilense* Sp7 (Fig. S5).

### 3.8 Adhesion to radish seeds and colonization of radish seedlings by *A. brasilense* strains

**Fig. 6:**
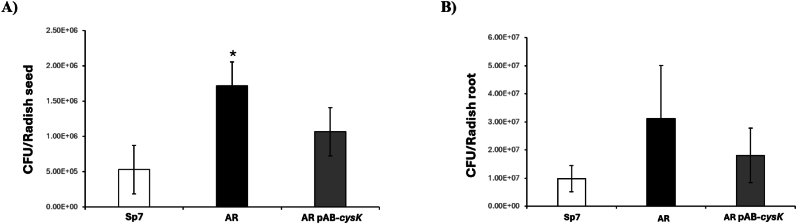
Adherence and colonization of *A. brasilense* strains on radish. (**A**) Adherence to radish seeds. Seeds were inoculated with approximately 5 × 10^7^ CFU/mL of the wild-type (*A. brasilense* Sp7), the *cysK*-A mutant (*A. brasilense* AR), or the complemented mutant (*A. brasilense* AR-pAB-*cysK*). Adhered bacteria were quantified 2 h post-inoculation. (**B**) Colonization of radish roots. Following inoculation, seeds were germinated, and the resulting seedlings were grown in sterilized soil for seven days. Bacteria colonizing the roots were quantified. Bar plots represent the mean CFU recovered ± standard deviation for each assay. An asterisk (∗) indicates a statistically significant difference between the mutant and the WT strain in the adherence assay (Student's t-test; p < 0.05). No significant differences were found in the root colonization assay.

Our previous data indicate that the *A. brasilense* AR mutant strain exhibits increased flocculation, exopolysaccharide synthesis, and biofilm formation when free-living. This led us to investigate whether the *cysK*-A mutant demonstrates altered adhesion to radish seeds compared to the wild-type strain, and whether it impacts the colonization of radish seedlings. To quantify adhesion to radish seeds, we performed a colony-forming unit (CFU) assay using *A. brasilense* Sp7, *A. brasilense* AR mutant, and *A. brasilense* AR-pAB-*cysK* complemented strains. Our findings showed that the *cysK*-A mutant strain adhered in a significantly greater proportion (1.03 × 10^7^ CFU/radish seed) than the wild-type strain (3.18 × 10^6^ CFU/radish seed). This difference was statistically significant (Student's t-test, *p* < 0.05). Importantly, this elevated adhesion was restored to wild-type levels when the mutant was complemented with the wild-type *cysK*-A gene (Fig. 6A).

**Fig. 7:**
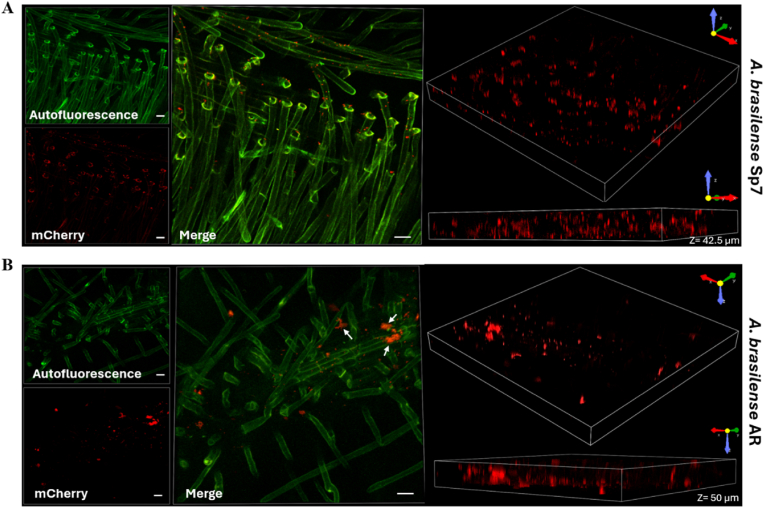
***A. brasilense* AR mutant forms thicker aggregates on radish roots than the wild-type strain.** Representative 3D CLSM images of radish roots 7 days post-inoculation. (**A**) Wild-type (WT) *A. brasilense* Sp7 (harboring pMP2449-5) displays a typical rhizosphere colonization pattern. (**B**) *cysK*-A mutant (*A. brasilense* AR, harboring pMP2449-5) forming visible, thicker, and denser aggregates (white arrows) compared to the WT. For both strains, bacteria are tagged with mCherry (red fluorescence), and the plant root autofluorescence is shown in green. Aggregate thickness was measured from the mCherry channel Z-stacks, revealing a depth of 42.5 μm for the WT and 50 μm for the AR mutant. Images were captured with a 20x objective, 2.0x digital zoom, and steps of 0.5 μm. Scale bar = 25 μm. (For interpretation of the references to color in this figure legend, the reader is referred to the Web version of this article.)

For radish plant colonization, CFU count assays at 7 days post-inoculation did not reveal statistically significant differences among the three strains (wild-type, *cysK*-A mutant, and complemented) (Fig. 6B). However, confocal microscopy analysis revealed that the *A. brasilense* AR mutant strain formed larger bacterial aggregates compared to the wild-type strain. These data suggest that the *A. brasilense* AR mutant strain exhibits increased aggregation even when associated with radish plants, reflecting its free-living behavior (Fig. 7). Despite the enhanced adhesion to radish seeds, this phenotype did not translate into altered overall colonization levels. This discrepancy may be attributable to a growth defect in the *A. brasilense* AR strain, likely provoked by cysteine deficiency.

### 3.9 Bioinformatic analyses of *cysE* and *cysK* genes

To investigate the lack of cysteine auxotrophy in the *cysK*-A mutant, we searched the *A. brasilense* Sp7 genome for homologs of CysK (ABB88852.1) (Table S1) and CysE (Table S2) proteins. To identify probable CysE homologs, we performed sequence homology searches using reported CysE amino acid sequences from *Brucella abortus* (WLU33920.1) and *Mycobacterium tuberculosis* (AYP16639.1). When the *B. abortus* CysE sequence was used as the query, we identified two putative CysE sequences codified in *A. brasilenses* Sp7 genome. The first hit showed 47.3 % identity and >90 % coverage and was designated as CysE-1. The second hit showed 38.2 % identity and 62 % coverage, designed as CysE-2. The two identified *A. brasilense* sequences share 42 % identity with 95 % coverage. Conversely, when the *M. tuberculosis* CysE sequence was used, it shared 45.2 % identity with CysE-1 and 46.7 % identity with CysE-2 of *A. brasilense* (Figs. S6 and S7).

A similar BLASTP search for CysK homologs identified two additional sequences. The first, showing 38 % identity and >90 % coverage, was predicted to be a second cysteine synthase and was designated as CysK*-*B. The second hit (21.82 % identity) was annotated as an l-cysteine desulfhydrase (Table S3).

To confirm that these are distinct loci, we analyzed the genomic context for each identified *cysE* and *cysK* homolog. The *cysE*-1 gene is flanked by a Glycosyl transferase upstream and a Transcriptional regulator downstream. The *cysE*-2 gene is flanked by a Homocitrate synthase upstream and a Nitrogen fixation protein NifW downstream. The *cysK*-A gene is flanked by a Rfr2 family transcriptional regulator and a hypothetical protein downstream. Finally, *cysK*-B is flanked by an ADP ribose pyrophosphatase upstream and an Alanine-tRNA(pro)hydrolase downstream (Fig. S8). These data suggest that there are two putative cysteine biosynthesis pathways from serine, codified in *A. brasilense* Sp7 genome.

Continuing the bioinformatic analysis, three-dimensional models of the CysK-A, and CysK-B proteins were generated using AlphaFold-3 (Fig. S9) and validated (Figs. S10 and S11, respectively). Comparison with known CysK-A crystal structures revealed that while CysK-B conserves essential motifs (TSGNT loop and GAG/GTGGT regions) (Fig. S12), but interestingly it also possesses unique structural features. Notably, we identified two large insertions and key amino acid changes, including a substitution at position 98 (M98Q) and an insertion of a tyrosine at position 127, relative to the canonical CysK-A sequence of *A. brasilense* Sp7 (Figures S12, S13 and S14).

The structural differences observed in CysK-B, particularly the M98Q substitution and Y127 insertion, suggest it may function differently from the canonical CysK-A. These changes are analogous to those in the CysK protein of *Brucella abortus*, which is regulated independently of CysE. In *B. abortus*, residues Gln96 and Tyr124 (corresponding to *A. brasilense* CysK-B Gln98 and Tyr127) are thought to occupy the active site pocket, sterically hindering the binding of the CysE terminal tail [38]. Therefore, it is plausible that CysK-B represents a CysE-independent cysteine synthase in *A. brasilense*, providing a redundant pathway for cysteine synthesis and explaining the viability of the *cysK-A* mutant.

## 4 Discussion

Our previous research on the *cysK*-A gene established its role in encoding O-acetylserine (thiol)-lyase (CysK-A), an enzyme vital for the final step of cysteine biosynthesis [13]. Despite the lack of cysteine auxotrophy in minimal medium, this suggested functional redundancy or alternative pathways for cysteine synthesis in *A. brasilense* Sp7. A comprehensive analysis of the *A. brasilense* AR *cysK*-A mutant has since revealed a multifaceted phenotype, highlighting the critical role of cysteine biosynthesis in various physiological processes, including flocculation, biofilm formation, oxidative stress, and plant adhesion. This current study provides compelling evidence that a functional CysK-A protein is indispensable for optimal growth, tolerance to oxidative stress, biofilm formation, adhesion, and colonization fitness. The observation that the *cysK*-A mutant exhibited a reduced final cell density in minimal media demonstrates that CysK-A is essential for optimal growth yield in *A. brasilense* Sp7. This growth impairment is fully rescued by genetic and chemical complementation, underscoring the importance of *de novo* cysteine synthesis for cellular proliferation and metabolic efficiency. Cysteine is a fundamental amino acid for protein biosynthesis and a precursor for essential sulfur-containing compounds, including cofactors and secondary metabolites [[4], [5], [6]]. Reduced cysteine availability in the *cysK*-A mutant likely impacts protein synthesis rates and the efficient functioning of metabolic pathways, leading to the observed growth defect. This finding is consistent with studies in other bacteria where disruption of cysteine biosynthesis pathways similarly impairs growth under various conditions. For instance, *cysE* and *cysB* mutants (encoding O-acetyl-serine sulfhydrylase and a transcriptional regulator, respectively) in *E. coli* and *Salmonella enterica* serovar Typhimurium have been reported to be unable to synthesize the l-cysteine precursor O-acetyl-l-serine. Consequently, these mutants will not grow on inorganic sulfur sources unless this product is provided [7]. Similarly, in *Brucella ovis*, genetic disruptions of cysteine biosynthesis genes lead to significant defects in growth, stress survival, and colonization fitness within host cells [8].

A striking phenotype of the *A. brasilense cysK*-A mutant was its enhanced flocculation and biofilm formation under free-living conditions. This hyper-aggregative behavior, along with increased biofilm mass, was consistently observed in different minimal media and was reverted upon wild-type *cysK*-A gene complementation. These findings strongly suggest that cysteine deficiency might act as a physiological signal, triggering a shift towards a sessile lifestyle or biofilm [11]. Biofilm formation is a complex process often associated with environmental stress, nutrient limitation, and host colonization strategies [3]. Cysteine biosynthetic genes have been shown to play roles in the biofilms formed by other bacteria, including *E. coli* and *Providencia stuartii* [11]. In these microorganisms, cysteine appears to negatively regulate biofilm formation, as mutations in *cysE* enhance biofilm formation and cause higher biomass production. The increased biofilms observed in the *E. coli cysE* mutant could be reduced by the exogenous addition of *O*-acetylserine (OAS), the product of CysE [11]. However, the underlying mechanism and whether OAS is the natural signal controlling biofilm formation remains unknown.

In *Vibrio fischeri*, genes necessary for biofilm formation have been identified through screening for mutants that failed to form wrinkled colonies, a type of biofilm. Several mutants exhibited defects in genes required for cysteine metabolism, including *cysH*, *cysJ*, *cysK*, and *cysN*. The *cysK* mutant displayed the most severe wrinkling defect. This defect could be complemented with a wild-type copy of the *cysK* gene, which encodes O-acetylserine sulfhydrylase, or by supplementing the medium with additional cysteine [10]. Notably, other mutants defective in cysteine biosynthetic genes did not negatively impact wrinkled colony formation, suggesting a specific role for CysK. CysK did not appear to control the activation of Syp regulators or transcription of the *syp* locus, but it did influence the production of the Syp polysaccharide. The *cysK* mutant also exhibited a defect in pellicle production that could be complemented by the *cysK* gene but not by cysteine, suggesting that, under these conditions, CysK is important for more than just cysteine production. These data reveal a critical role for *cysK* in symbiotic colonization by *V. fischeri* [10].

The observed increase in exopolysaccharide production, particularly β-1,3 and β-1,4 linked glycans as indicated by enhanced Calcofluor binding, provides a molecular explanation for the increased aggregation and biofilm thickness. EPS are key components of the biofilm matrix, providing structural integrity, protecting cells from environmental insults, and facilitating surface adherence [39]. The hyper-aggregative phenotype, characterized by increased biofilm thickness and notable EPS production, suggests that cysteine limitation induces a shift towards a sessile lifestyle. This adaptive response likely enables *A. brasilense* Sp7 to survive periods of metabolic stress [1,40]. Such stress-induced shifts in lifestyle are well-documented in other bacteria, where nutrient deprivation often promotes biofilm formation and persistence. For Instance, in *Bacillus subtilis*, serine starvation causes ribosomes to pause on specific serine codons, leading to a decrease in the translation rate of *sinR* gene, which encodes a master repressor for biofilm matrix genes, ultimately triggering biofilm induction [41]. Similarly, in *Pseudomonas aeruginosa*, several amino acids (arginine, ornithine, isoleucine, leucine, valine, phenylalanine, and tyrosine) promote robust biofilm formation and reduce swarming motility [42]. In *Pseudomonas putida*, arginine and its precursor aspartic acid have divergent effects on biofilm formation; increasing concentrations of arginine stimulate surface attachment, while aspartic acid causes the opposite effect [43].

Our data strongly indicate that the observed increase in biofilm formation and exopolysaccharide synthesis in the *A. brasilense cysK*-A mutant is likely mediated by elevated intracellular levels of c-di-GMP. The distinct red coloration observed in the *cysK*-A mutant carrying the c-di-GMP biosensor plasmid (pFY4535) provides evidence for increased c-di-GMP. The c-di-GMP is a ubiquitous bacterial second messenger that profoundly influences the transition between planktonic and sessile lifestyles, primarily by regulating flagellar motility, EPS production, and biofilm formation [44]. Higher c-di-GMP levels generally promote biofilm formation and EPS synthesis while repressing motility. Our results propose that the loss of *cysK*-A gene and the probable concomitant decrease in cysteine synthesis directly or indirectly lead to increased c-di-GMP levels, thereby driving the observed changes in aggregation and biofilm architecture. The precise mechanism linking cysteine availability to c-di-GMP metabolism in *Azospirillum* deserves further investigation. It is plausible that altered metabolic flux or stress responses due to cysteine limitation could impact the activity of diguanylate cyclases (DGCs) or phosphodiesterases (PDEs), enzymes responsible for c-di-GMP synthesis and degradation, respectively [45].

Cysteine is a crucial precursor for glutathione (GSH) synthesis, a tripeptide that plays a central role in antioxidant defense and redox homeostasis in many bacteria [6]. We hypothesize that defects in cysteine biosynthesis would impair GSH synthesis and consequently increase susceptibility to oxidative stress. The *A. brasilense* AR mutant exhibited significantly increased sensitivity to exogenous H_2_O_2_ stress, as evidenced by a larger zone of inhibition in the disc diffusion assay. This heightened sensitivity was fully restored by *cysK*-A gene complementation, highlighting the contribution of CysK-A-dependent cysteine synthesis to H_2_O_2_ tolerance in *A. brasilense* Sp7. This finding aligns with studies in other microorganisms where impaired cysteine or GSH biosynthesis leads to increased susceptibility to various forms of oxidative stress (e.g., *Bacillus subtilis* [46]; *Mycobacterium tuberculosis* [47]). The inability to efficiently neutralize reactive oxygen species (ROS) likely contributes to the observed growth defect. It may also play a role in triggering stress-induced aggregative phenotypes [48].

The enhanced flocculation, EPS production, and biofilm formation observed in the free-living *A. brasilense* AR mutant suggested an altered interaction with its plant host. Indeed, the *A. brasilense* AR mutant demonstrated significantly greater adhesion to radish seeds compared to the wild-type strain. This increased adhesion is likely mediated by the overproduction of EPS, which enhances bacterial attachment to surfaces [49]. However, despite increased initial adhesion, the *cysK*-A mutation did not translate into statistically significant differences in overall radish plant colonization as assessed by CFU counts at seven days post-inoculation. Confocal microscopy, however, revealed that the *cysK*-A mutant formed larger bacterial aggregates on radish roots, mirroring its free-living aggregative behavior. This suggests that while the *cysK*-A mutant can adhere and colonize, its mode of colonization involves enhanced aggregation, potentially impacting nutrient exchange or other beneficial interactions with the host in ways not captured by CFU counts alone. The lack of a significant difference in overall colonization despite increased adhesion and aggregation might be attributable to the growth defect provoked by cysteine deficiency. It is also possible that the increased adherence to the root could also be non-specific, since the mutant is generally sticky. Further research is needed to dissect the specific roles of *cysK*-A-mediated cysteine synthesis in different stages of *Azospirillum*-plant interaction.

Bioinformatics analysis provided crucial context for physiological observations. The *A. brasilense* Sp7 genome encodes two putative CysE proteins and one additional putative CysK protein (CysK-B). We propose that the two CysE proteins represent full-length and truncated isoforms, respectively. This distinction is critical because CysE, which catalyzes the rate-limiting step in *de novo* cysteine biosynthesis, is maximally active in its hexameric conformation or full-length. This conformation is achieved via a trimer-trimer interaction mediated by the N-terminus. The truncated CysE variant, which lacks approximately 76 N-terminal amino acids, is thought to be incapable of this hexameric formation, resulting in a less active trimeric assembly [50]. We hypothesize that only the full-length CysE isoform can interact with CysK-A to form the cysteine synthase complex, while the truncated isoform, despite likely retaining partial SAT activity, cannot.

Identification of the additional putative CysK protein, CysK-B, suggests redundant cysteine biosynthesis capabilities in *A. brasilense* Sp7. CysK-B shares 38 % identity with CysK-A, but their structural differences are particularly intriguing: CysK-B features two key insertions and significant amino acid substitutions (M98Q and a Y127 insertion). These structural variations suggest CysK-B may be a distinct cysteine synthase variant, potentially regulated by a different CysE mechanism. This hypothesis is supported by findings in *Brucella abortus* [38], where analogous substitutions (Gln96 and Tyr 124) in the active site pocket were shown to abrogate the interaction with the CysE C-terminal tail, leading to CysE-independent internal regulation. This parallel suggests that CysK-B in *A. brasilense* Sp7 is also subject to an alternative cysteine biosynthesis pathway, which would explain the residual cysteine prototrophy observed in the *cysK*-A mutant.

Future studies should experimentally validate the enzymatic activity of CysK-B and investigate its contribution to cystine homeostasis, particularly under *in plant* conditions where different metabolic demands might activate alternative pathways. The interplay between CysK-A and CysK-B, and their respective regulatory networks, likely contributes to the metabolic adaptability and robust lifestyle of *A. brasilense* Sp7.

## 5 Conclusions

This study reveals that the *A. brasilense* CysK-A enzyme, responsible for the final step of cysteine biosynthesis, plays a surprisingly complex and central role in the physiology of *A. brasilense* Sp7. Although not essential for survival in minimal media. The loss of a functional *cysK*-A gene triggers a cascade of effects, impairing growth and oxidative stress tolerance while paradoxically enhancing aggregation, biofilm formation, and initial plant adhesion. These phenotypes are likely interconnected and orchestrated by the second messenger c-di-GMP, highlighting how a metabolic limitation can trigger a profound shift in bacterial lifestyle from planktonic to sessile.

## CRediT authorship contribution statement

**Job Herrera-Galindo:** Writing – original draft, Software, Methodology, Investigation, Formal analysis. **Clara Andrea Alcantara-Rosales:** Methodology, Investigation, Formal analysis. **Oscar Mateo Ojeda:** Software, Methodology, Investigation, Formal analysis, Data curation, Conceptualization. **María Luisa Xiqui-Vázquez:** Validation, Supervision, Project administration, Methodology, Formal analysis, Data curation. **Claudia Mancilla-Simbro:** Writing – review & editing, Methodology, Investigation, Formal analysis, Data curation, Conceptualization. **Sandra Reyes-Carmona:** Writing – review & editing, Investigation. **Beatriz Eugenia Baca:** Writing – review & editing, Visualization, Investigation, Funding acquisition, Conceptualization. **Alberto Ramírez-Mata:** Writing – review & editing, Visualization, Validation, Project administration, Investigation, Funding acquisition, Formal analysis, Data curation, Conceptualization.

## Ethical approval and consent to participate

Not applicable, No human samples or clinical data were used in this study.

## Consent for publication

All authors approved the final version of the manuscript.

## Declaration of competing interest

The authors declare that they have no known competing financial interests or personal relationships that could have appeared to influence the work reported in this paper.

## Data Availability

No data was used for the research described in the article. **Zenodo** Azospirillum brasilense Biofilm formation analyzed by confocal microscopy (Original data)
