# Supplementary material for: Inactivation of Cysteine Synthase CysK-A enhances flocculation, biofilm formation, and sensitivity to oxidative stress in Azospirillum brasilense Sp7
Source: Biofilm. 2025 Dec 12;11:100335. doi: 10.1016/j.bioflm.2025.100335 (PMC12811640; doi:10.1016/j.bioflm.2025.100335)
Supplement: Multimedia component 1 [file mmc1.docx]

**Supplementary data**

**Inactivation of Cysteine Synthase CysK-A enhances flocculation, biofilm formation, and sensitivity to oxidative stress in *Azospirillum brasilense* Sp7.**

**Table S1.** Sequences used for bioinformatic analysis and accession numbers.

| **Protein** | **Gene** | **Accession number** | **Microorganism** |
| --- | --- | --- | --- |
| Cysteine synthase A | *cysK*-A | WP_035669937.1 | *Azospirillum brasilense* Sp7 |
| Cysteine synthase A | *cysK*-B | WP_035673815.1 | *Azospirillum brasilense* Sp7 |
| Cysteine synthase A | *cysK* | WP_002741039.1 | *Microcystis aeruginosa* |
| O-acetylserine sulfhydrylase | *cysK* | WP_003899275.1 | *Mycobacterium tuberculosis* |
| Cysteine synthase A | *cysK* | WP_004684481.1 | *Brucella ovis* |
| Cysteine synthase A | *cysK* | WP_002964172.1 | *Brucella abortus* |

**Table S2.** Sequences used for bioinformatic analysis and accession numbers of CysE proteins

| **Protein** | **Gene** | **Accesion number** | **Query Cover** | **Microorganism** |
| --- | --- | --- | --- | --- |
| Serine  *O*-acetyltransferase | *cysE*-1 | WP_014199849.1 | 90% | *Azospirillum brasilense* Sp7 |
| Serine  *O*-acetyltransferase | *cysE*-2 | ALJ34749.1 | 62% | *Azospirillum brasilense* Sp7 |
| Serine  *O*-acetyltransferase | *cysE* | WLU33920.1 | 100% | *Brucella abortus* |
| Serine  *O*-acetyltransferase | *cysE* | AYP16639.1 | 100% | *Mycobacterium tuberculosis* |

**Table S3.** BLASTP search results from *A. brasilense* Sp7 genome information showing the three protein sequences localized.

| **Description** | **Scientific Name** | **Query Cover** | **E-value** | **Percentage of identity** |
| --- | --- | --- | --- | --- |
| WP_035669937.1 cysteine synthase A  (CysK-A) | *A. brasilense* Sp7 | 100% | 0 | 99.69% |
| WP_035673815.1 cysteine synthase A  (CysK-B) | *A. brasilense* Sp7 | 95% | 9.00E-60 | 38.73% |
| WP_035683056.1 PLP-dependent cysteine synthase family protein | *A. brasilense* Sp7 | 92% | 2.00E-07 | 21.82% |


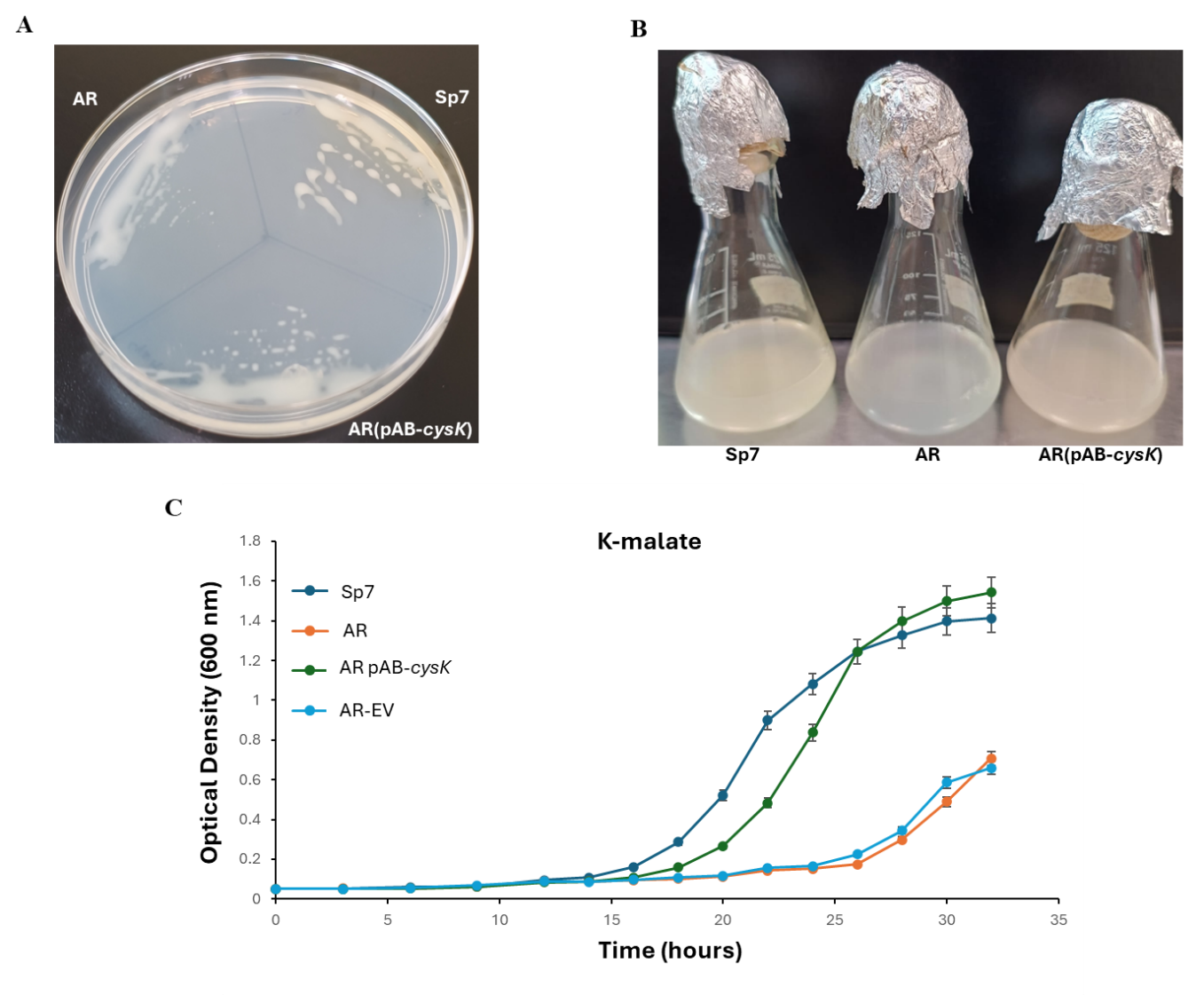


**Figure S1.** Inactivation of *cysK*-A impairs the growth of *A. brasilense*, a defect rescued by genetic complementation. Growth of the wild-type (*A. brasilense* Sp7), the *cysK-A* mutant (*A. brasilense* AR), the complemented strain (*A. brasilense* AR-pAB-*cysK*), and the empty vector control (*A. brasilense* AR-EV) was assessed on solid and in liquid minimal media. (**A**) Representative growth on K-malate agar plate after 72 hours of incubation. (**B**) Endpoint growth in K-lactate liquid medium after 48 hours. (**C**) Growth curves in liquid minimal medium containing malate as the carbon source. In the graphs, strains are represented as follows: wild-type *A. brasilense* Sp7 (dark blue), *A. brasilense* AR (orange), *A. brasilense* AR-pAB-*cysK* (green), and *A. brasilense* AR-EV (light blue). Error bars represent the standard deviation of technical replicates from a representative experiment.


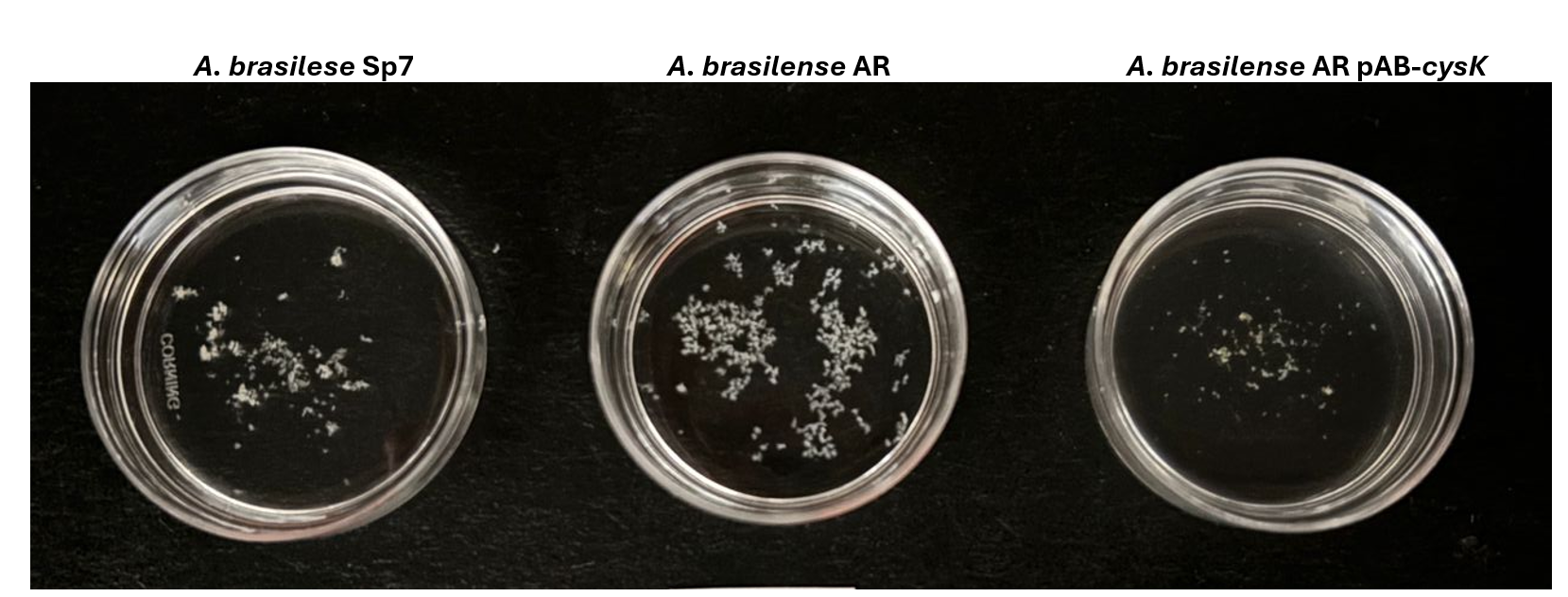


**Figure S2.** Flocculation phenotype of *A. brasilense* strains. Photographs show cultures of the wild-type (*A. brasilense* Sp7), the *cysK-A* mutant (*A. brasilense* AR), and the complemented strain (*A. brasilense* AR-pAB-*cysK*) after 24 hours of incubation in flocculation medium.


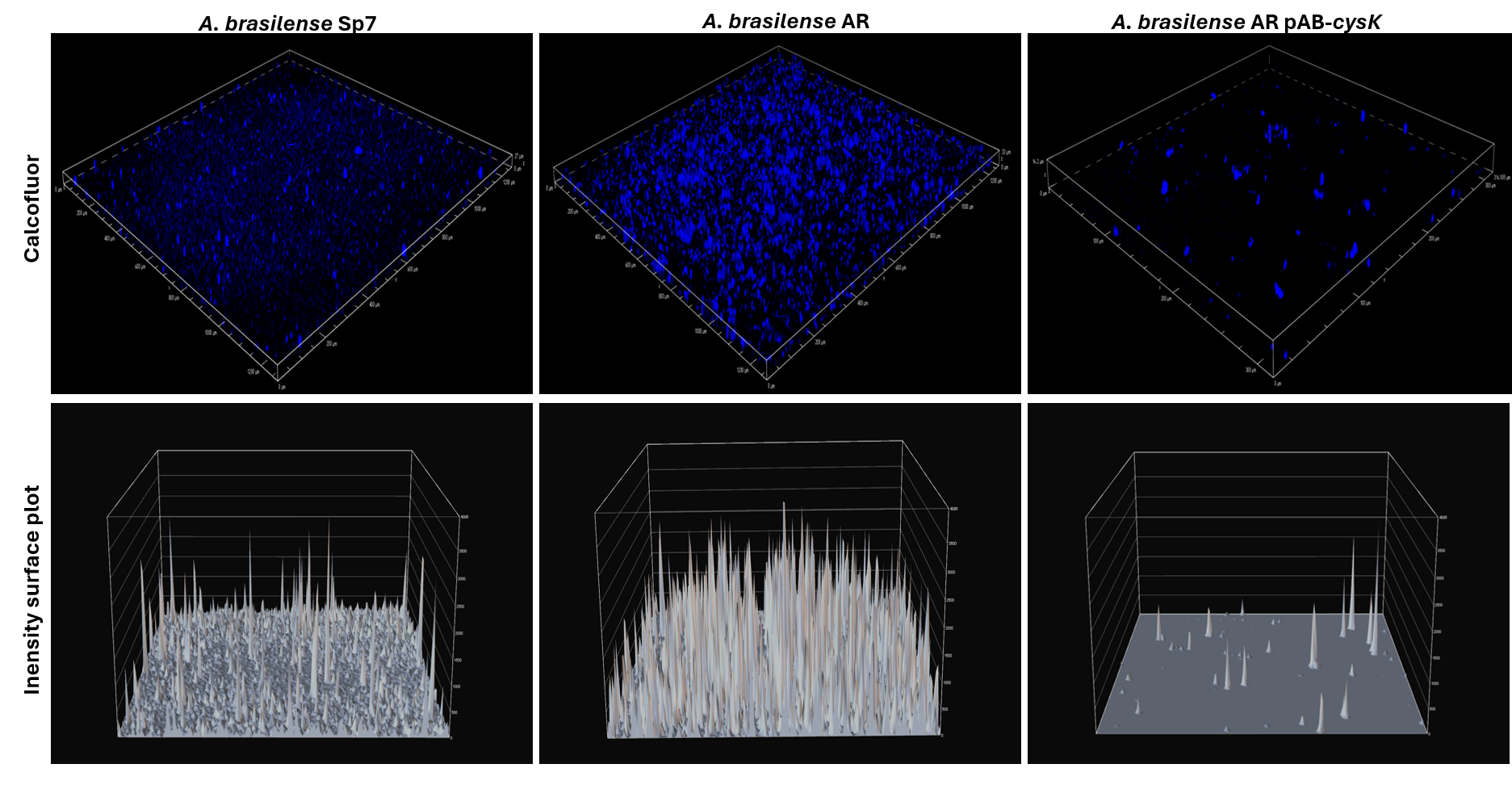


**Figure S3.** Biofilm formation analysis by confocal microscopy. Three-dimensional reconstructions of 5-day-old static biofilms formed by the wild-type (*A. brasilense* Sp7), the *cysK*-A mutant (*A. brasilense* AR), and the *cysK*-A mutant complemented (*A. brasilense* AR-pAB-*cysK*) strains. Biofilms were grown on liquid Nfb* medium and stained with Calcofluor (85 µM) to visualize β-linked polysaccharides (blue fluorescence). For each strain, an intensity surface plot (gray) is shown to illustrate the distribution and density of the fluorescent signal. All images were acquired with a 20x objective using identical confocal microscopy settings for laser intensity, contrast, and zoom. The images are representative of at least three independent experiments.


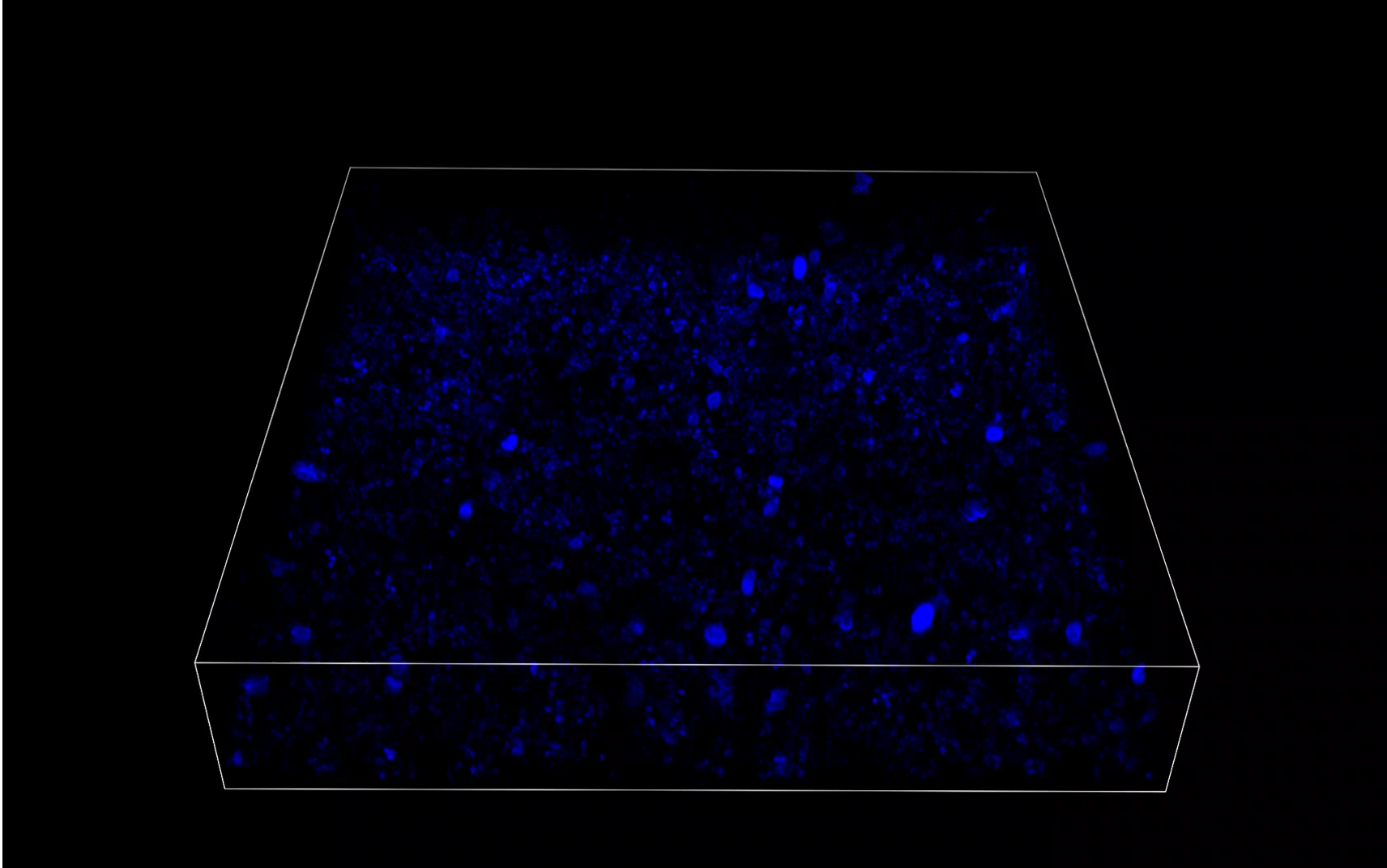


**Video 1. Three-dimensional (3D) CLSM reconstruction of an *A. brasilense* Sp7 biofilm.** The biofilm was grown for 5 days at 30°C in Nfb* medium supplemented with 85 µM Calcofluor. The 3D reconstruction was generated from images captured at 60x magnification using a Nikon Eclipse Ti-E C2+ microscope. The video was assembled using Nikon NIS Elements software.


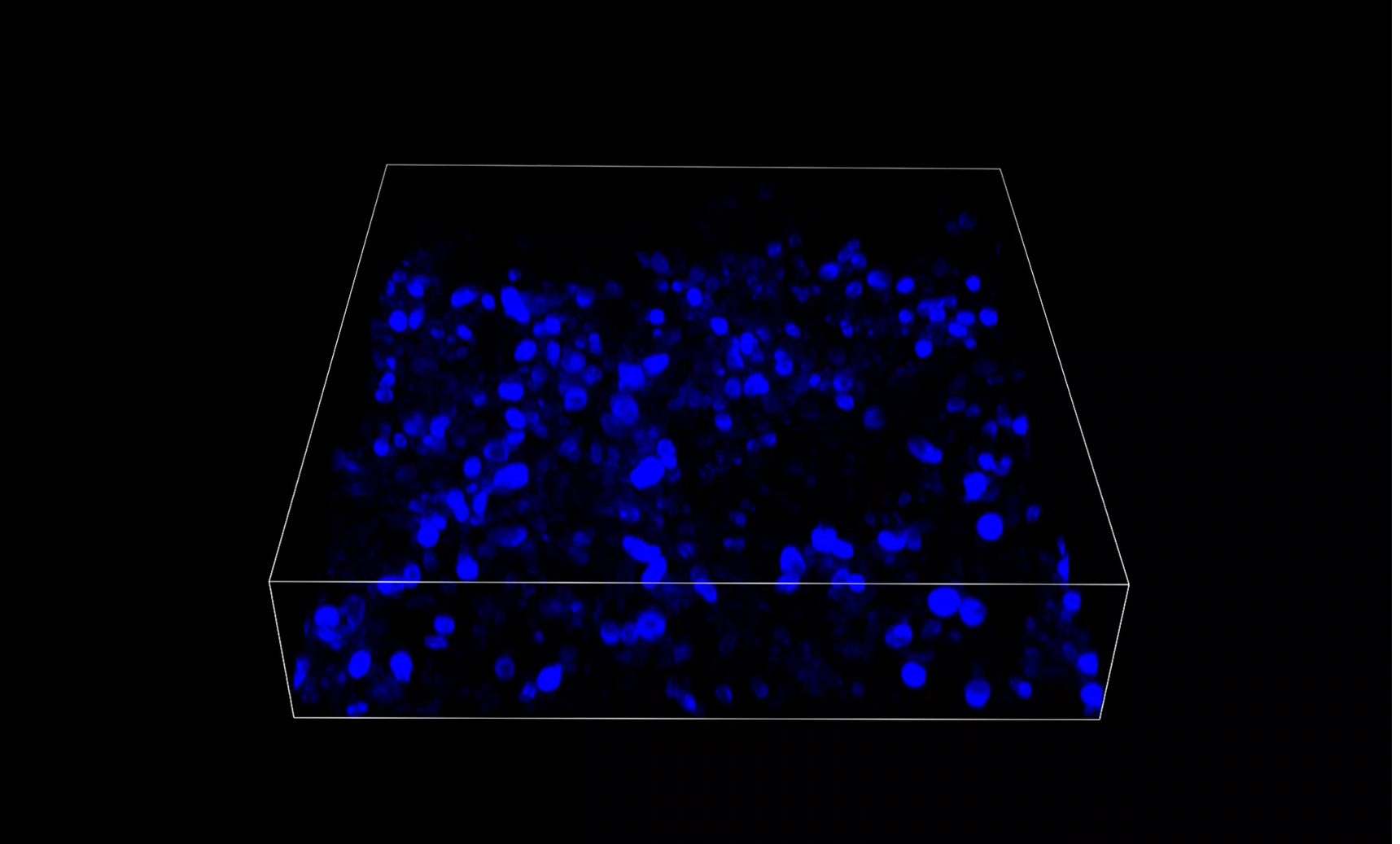
**Video 2**. **Three-dimensional (3D) CLSM reconstruction of an *A. brasilense* AR biofilm.** Biofilm was grown for 5 days at 30°C in Nfb* medium supplemented with 85 µM Calcofluor. The blue fluorescence, corresponding to Calcofluor staining, highlights structures resembling cyst-like cells within *cysK*-A mutant biofilm. The 3D reconstruction was generated from images captured at 60x magnification using a Nikon Eclipse Ti-E C2+ microscope. The video was assembled using Nikon NIS Elements software.


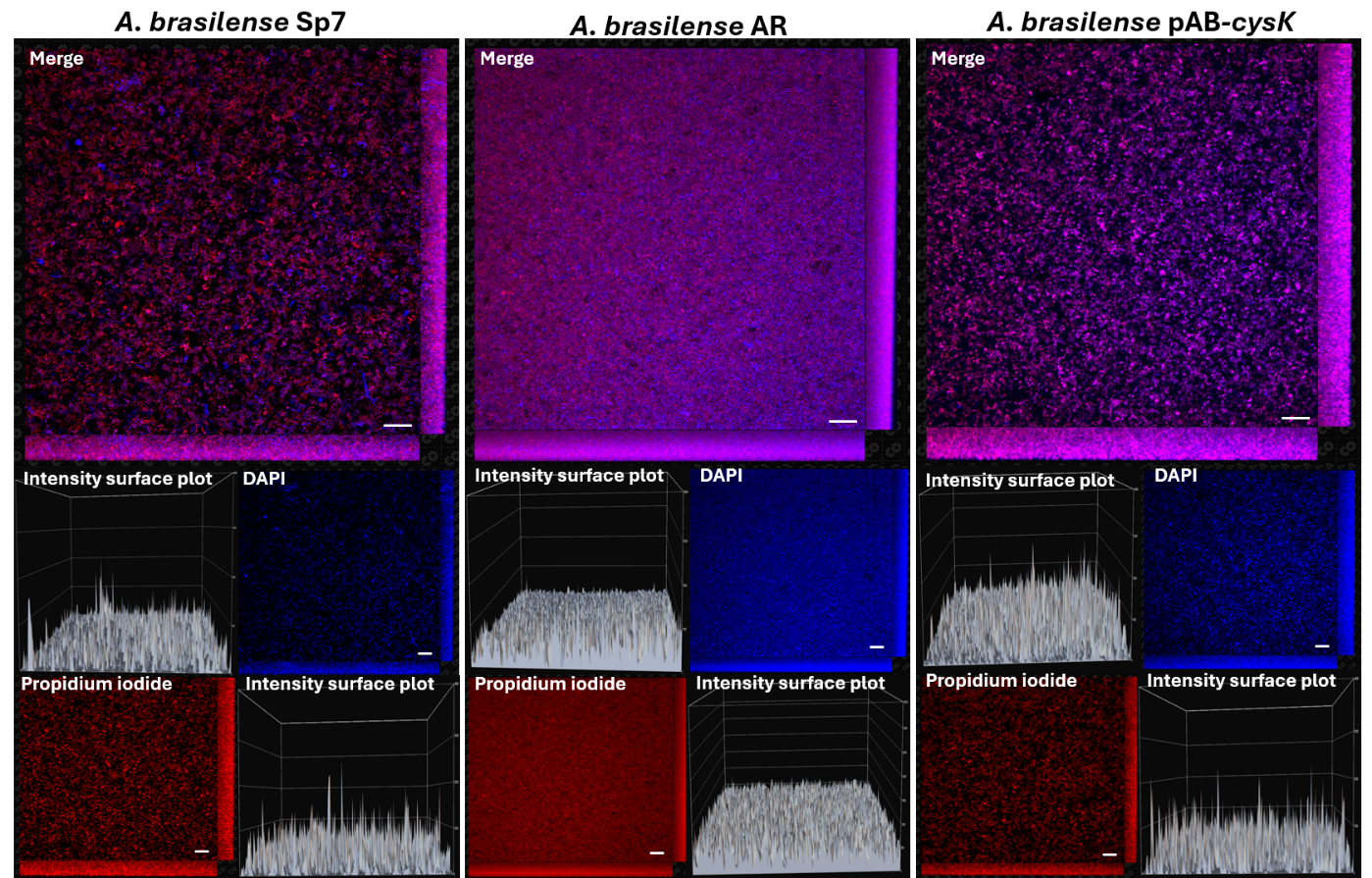


**Figure S4. Orthogonal CLSM views and 3D Surface plots of *A. brasilense* biofilms to analyze cell viability.** Representative images of five-day-old biofilms formed by *A. brasilense* Sp7, *A. brasilense* AR, and the complemented strain *A. brasilense* AR-pAB-*cysK* in Nfb* liquid medium. Cultures were grown in a fluorodish and stained with DAPI (blue) and propidium iodide (red). The central panels show horizontal (x-y) optical sections, flanked by vertical (x-z and y-z) sections. The corresponding intensity surface plot for each channel is shown in gray. Scale bar = 10 µm.


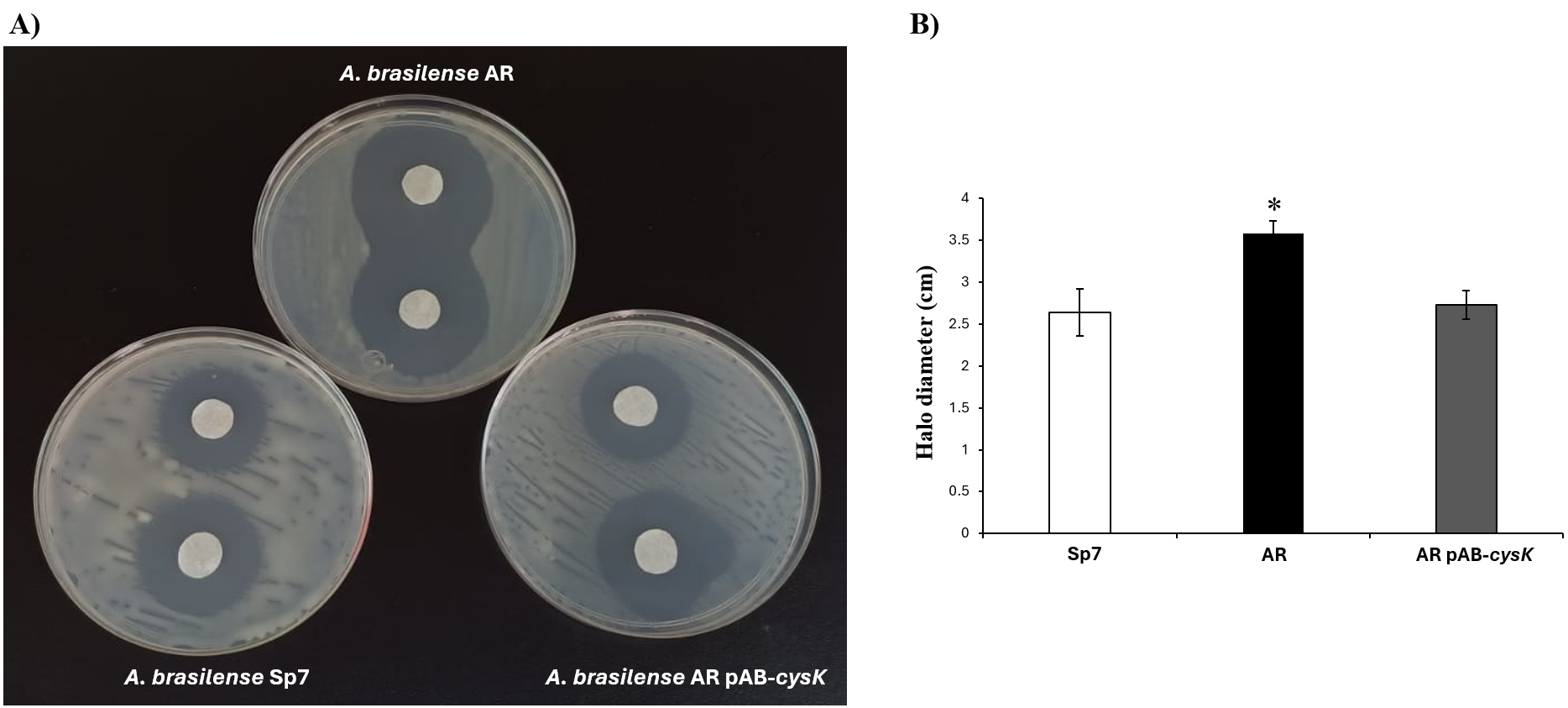


**Figure S5.** Sensitivity of *A. brasilense* strains to hydrogen peroxide (H_2_O_2_). A disk diffusion assay was used to assess sensitivity. Bacterial lawns of the WT strain *A. brasilense* Sp7, *cysK*-A mutant *A. brasilense* AR, and the complemented *A. brasilense* AR-pAB*-cysK* strain were exposed to a paper disk containing 10 µl of 1% H_2_O_2_. (**A**) Representative images showing the resulting zones of inhibition (halos) for each strain. (**B**) Quantification of the halo diameters. Data are presented as the mean diameter (cm) ± standard error from five independent experiments. An asterisk (*) indicates a significant difference compared to the WT strain (Student’s t-test, *p* < 0.01).


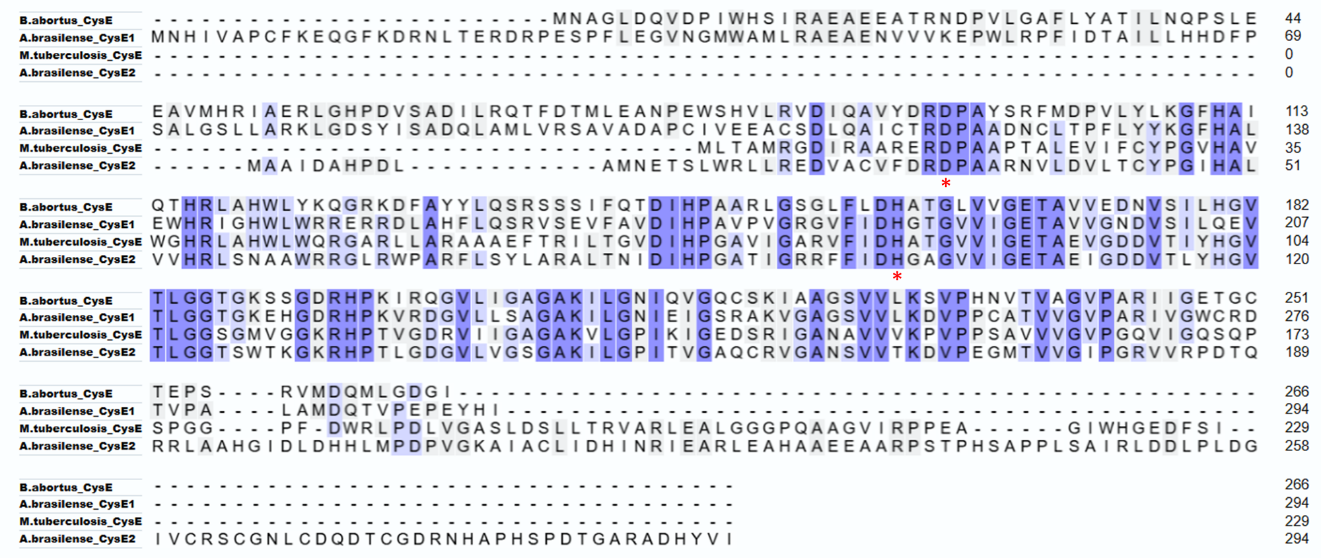


**Figure S6.** Multiple sequence alignment of CysE bacterial isoforms. Fully conserved residues are highlighted in dark blue, and partially conserved residues are in light blue. Catalytic dyad residues Aspartic acid (D) and Histidine (H) are indicated by a red asterisk (*). The alignment includes the following sequences: *A. brasilense* Sp7 CysE-1 (WP_014199849.1) and CysE-2 (ALJ34749.1); *B. abortus* CysE (WLU33920.1); and *M. tuberculosis* CysE (AYP16639.1). The alignment was generated using Clustal Omega within the UniProt Align platform.


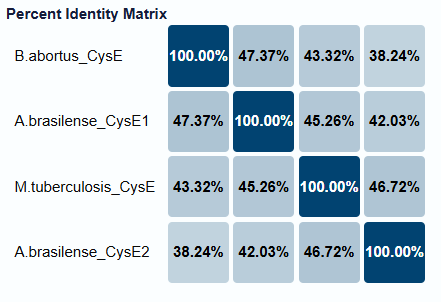


**Figure S7.** Percent identity matrix derived from a multiple sequence alignment of bacterial CysE isoforms. The following amino acid sequences were used for the alignment: *A. brasilense* Sp7 CysE-1 (WP_014199849.1) and CysE-2, (ALJ34749.1); *B. abortus* CysE (WLU33920.1); and *M. tuberculosis* CysE (AYP16639.1). The multiple sequence alignment was generated using Clustal Omega within the UniProt Align platform.


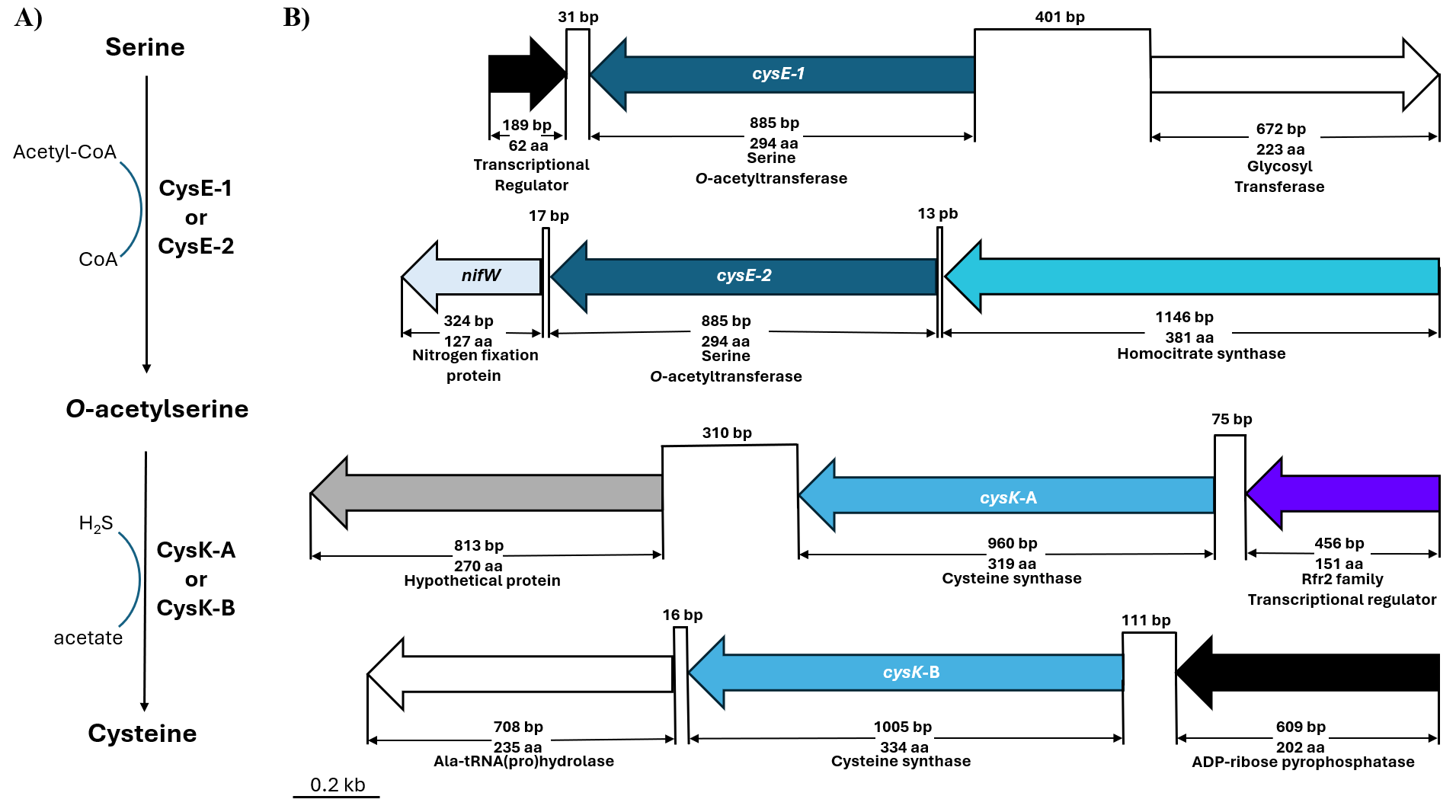


**Figure S8.** (**A**) Schematic of the cysteine biosynthesis pathway from serine. Black arrows indicate putative cysteine biosynthesis enzymes annotated in the *A. brasilense* Sp7 genome (RefSeq accession no. GCF_002027385.1). The enzymes shown are CysE (*O*-acetylserine transferase), represented by CysE-1 (WP_014199849.1; *cysE*-1) or CysE-2 (ALJ34749.1; *cysE*-2), and CysK (cysteine synthase), represented by CysK-A (WP_035669937.1 *cysK*-A), and CysK-B (WP_035669937.1 *cysK*-B). (**B**) Genomic organization of the *cysE* and *cysK* genes in *A. brasilense* Sp7 genome. Arrows represent genes and their direction of transcription. Spaces between arrows represent intergenic regions. The scale bar corresponds to 0.2 kb.

**CysK-A (AF-3) CysK (4LMA) CysK-B (AF-3) CysK (5JIS)**


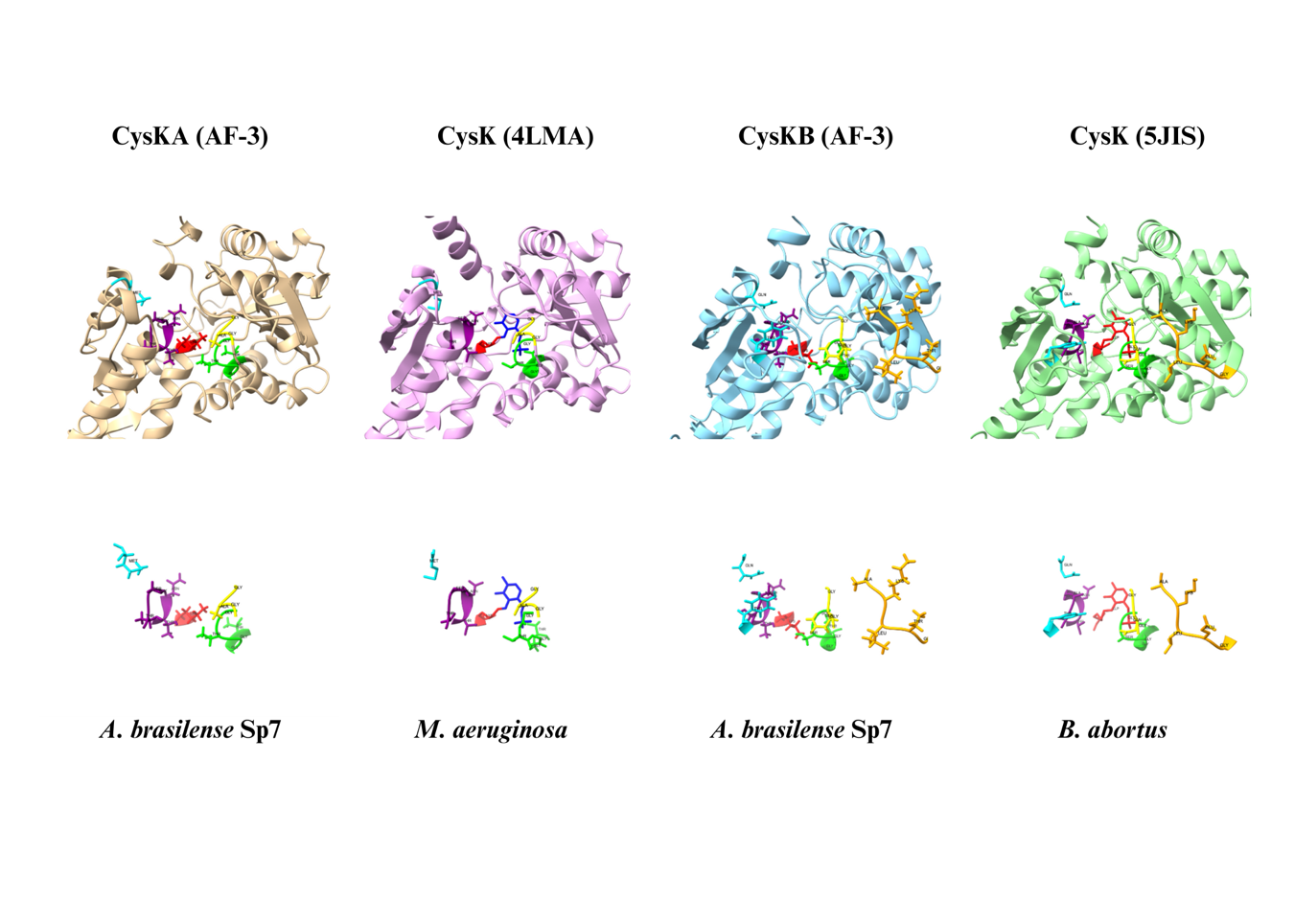


**Figure S9.** Comparison between crystallographic structures and AI-generated models. The three-dimensional predicted structures from *A. brasilense* Sp7 are displayed in brown (WP_035669937.1) and blue (WP_035673815.1). In addition, CysK from *Microcystis* *aeruginosa* is represented in pink (4LMA), while CysK from *Brucella abortus* is illustrated in green (5JIS). The interaction pocket of PLP is depicted in an array of colors corresponding to various essential motifs, including the catalytic lysine (red), TSGNT loop (purple), GAG region (yellow), and GT/SGGT region (green).


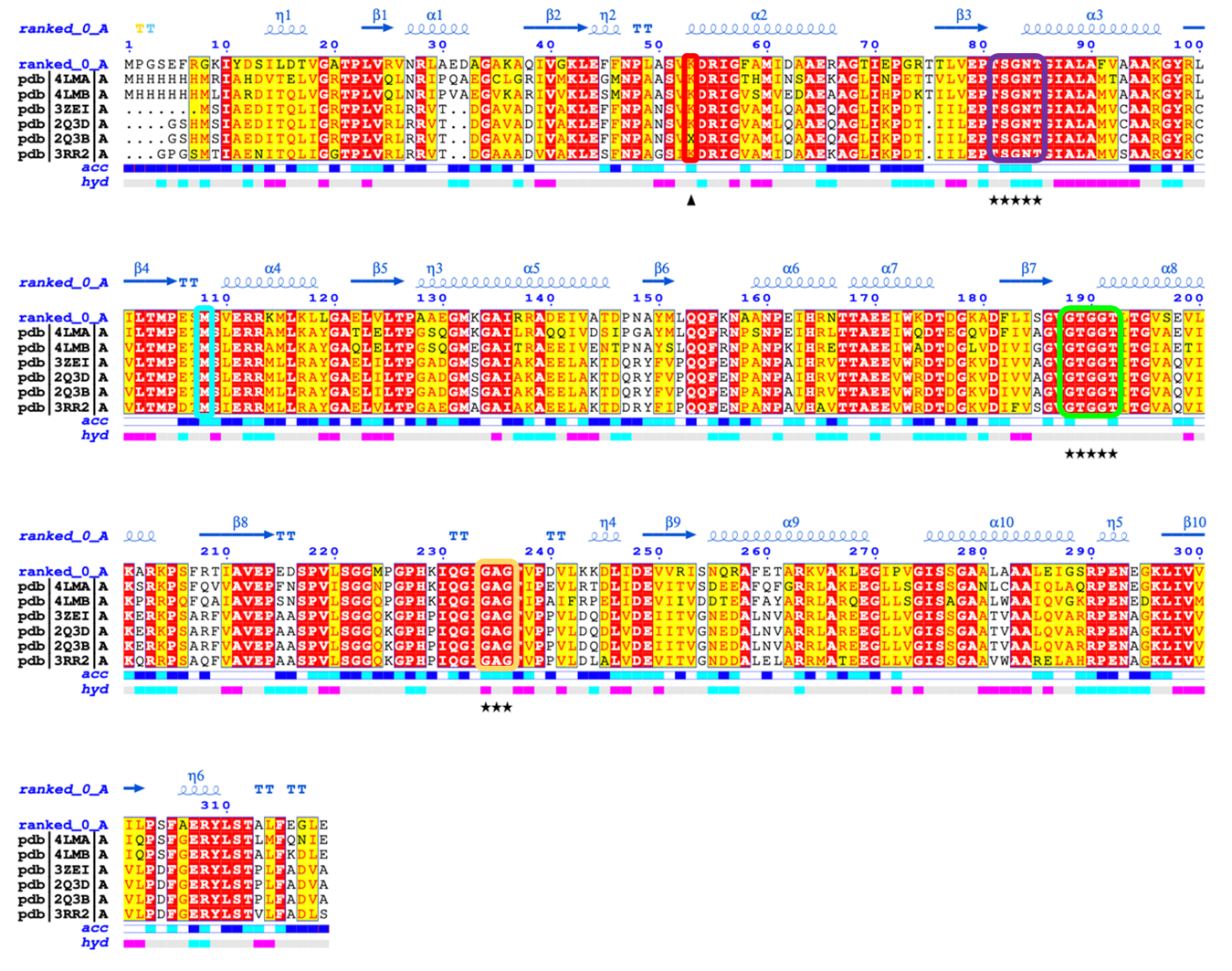


**Figure S10**. Foldscript v1.2 analysis of CysK-A protein and alignment with the PDB database. Conserved motifs are presented: catalytic lysine (red), TSGNT loop (purple), GAG region (yellow), and GT/SGGT region (green). Also, key amino acid differences are colored in cyan. **4LMA**, O-acetylserine sulfhydrylase CysK1 from *Microcystis aeruginosa.* **4LMB**, O-acetylserine sulfhydrylase CysK2 complexed with cystine from *Microcystis aeruginosa*. **3ZEI**, *Mycobacterium tuberculosis* O-Acetylserine Sulfhydrylase (OASS) CysK1 in complex with a small molecule inhibitor. **2Q3D**, O-Acetylserine Sulfhydrylase (OASS) From *Mycobacterium tuberculosis* in complex with the reaction intermediate Alpha-aminocrylate. **2Q3B**, O-Acetylserine Sulfhydrylase (OASS) Holoenzyme from *Mycobacterium tuberculosis*. **3RR2**, O-Acetylserine Sulfhydrylase (OASS from *Mycobacterium marinum.*


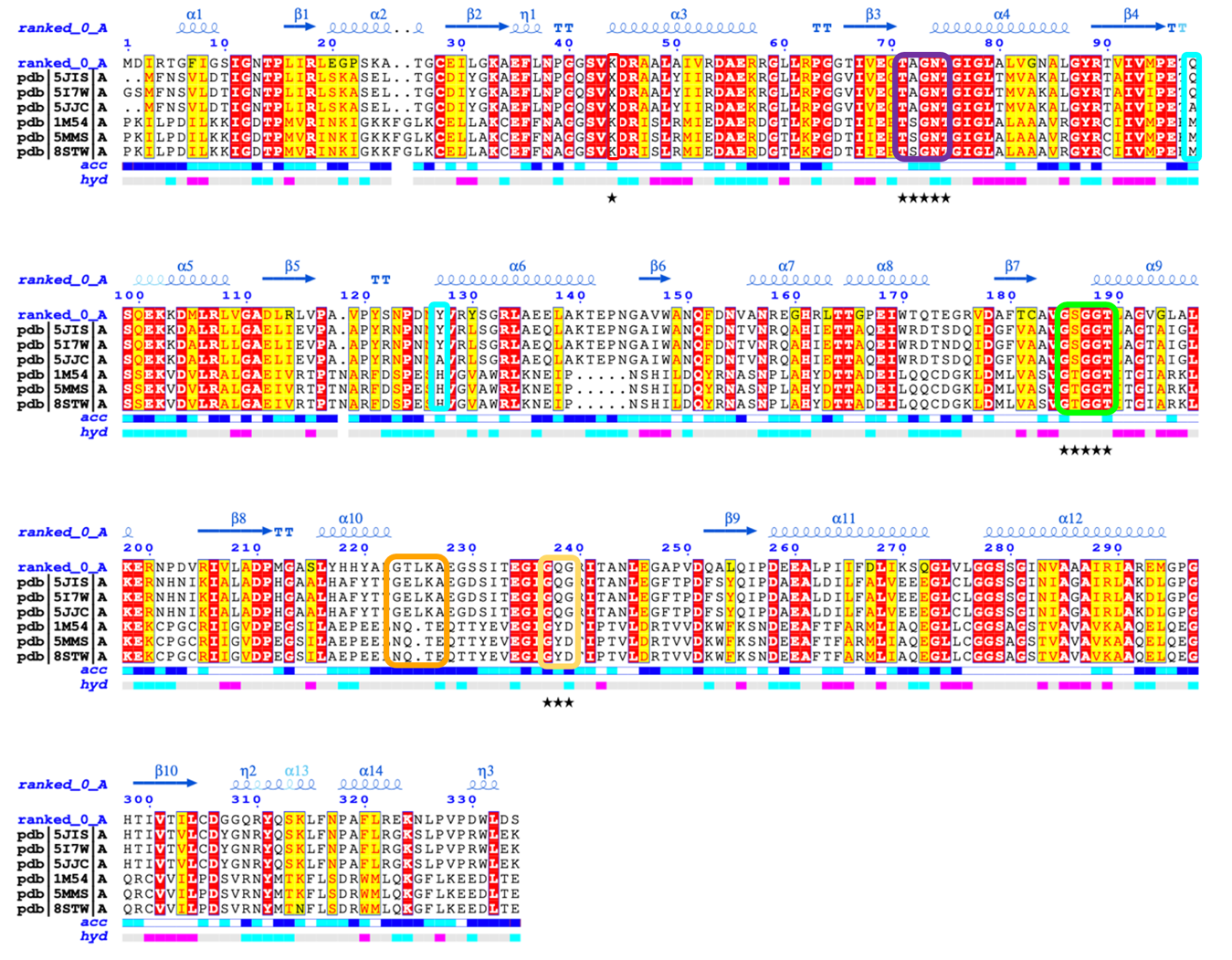


**Figure S12.** Foldscript v1.2 analysis of CysK-B protein and alignment with the PDB database. Conserved motifs are presented: catalytic lysine (red), TSGNT loop (purple), GAG region (yellow), and GT/SGGT region (green). Also, key amino acid differences are colored in cyan: M98Q and Y127 insertion. **5JIS**, O-acetyl serine sulfhydrylase from *Brucella abortus.* **5I7W**, Cysteine Synthase from *Brucella suis.* **5JJC**, double mutant (Q96A-Y125A) O-Acetyl Serine Sulfhydralase from *Brucella abortus.* **1M54**, Cystathionine-beta-synthase: reduced vicinal thiols. **5MMS**, Human cystathionine beta-synthase (CBS) p.P49L delta409-551 variant. **8STW**, K384N Human Cystathionine-beta-synthase (delta 411-551).

**
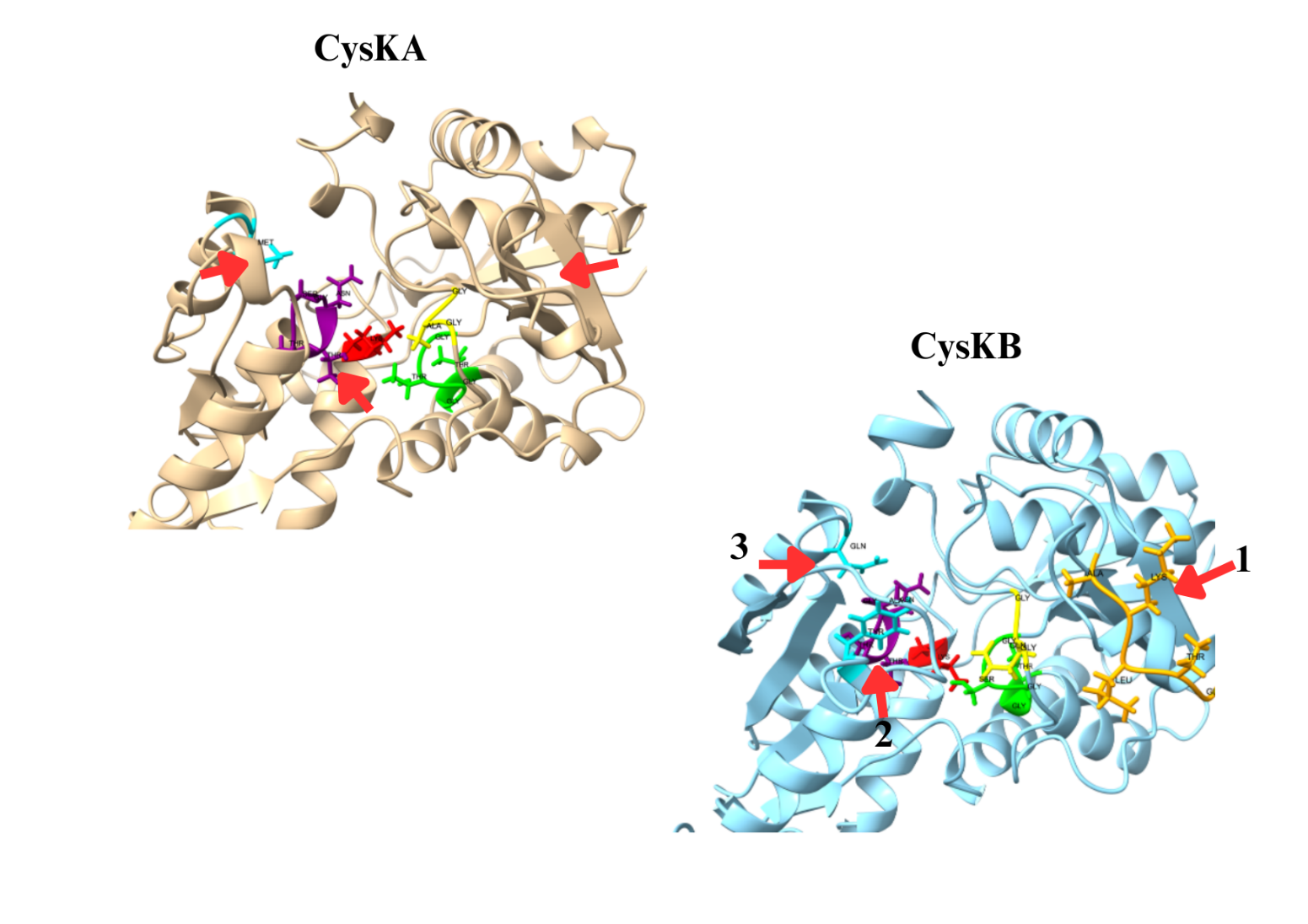
**

**CysK-B**

**CysK-A**

**Figure S12.** Key differences between CysK-A and CysK-B. The predicted structures are presented in brown (CysK-A) and blue (CysK-B), key differences are indicated with red arrows, including the (1) GTLKA insertion region, (2) the Y127 insertion, and the (3) M98Q amino acid change in CysK-B.


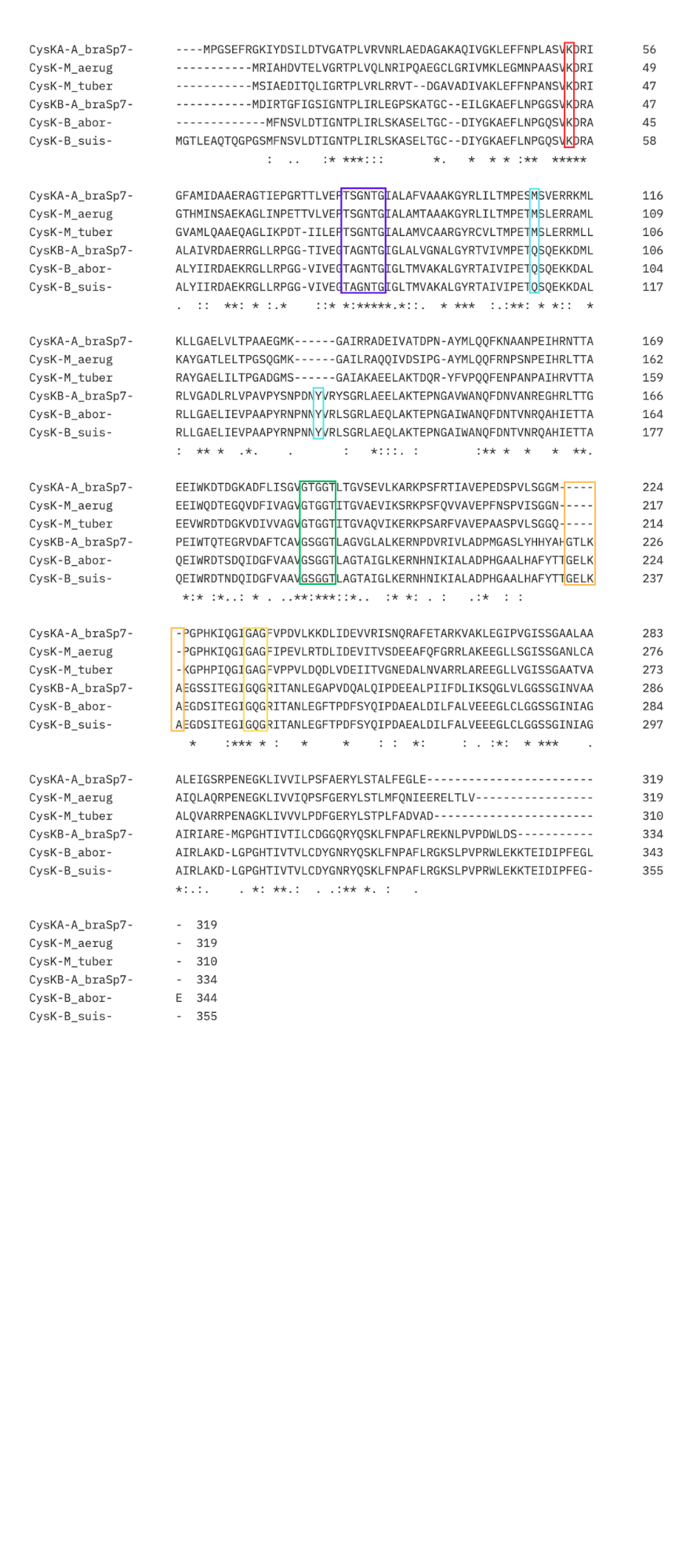


**Figure 13.** Protein sequence alignment between CysK homologous proteins. Interaction pockets of CysK-PLP and homologs are colored in a variety of colors corresponding to different necessary motifs, such as catalytic lysine (red), TSGNT loop (purple), GAG region (yellow), and GT/SGGT region (green) also key amino acid differences are colored in cyan M98Q and Y127 insertion, insertion regions are labeled with a blue arrow. Strains: *Azospirillum brasilense, Microcystis aeruginosa, Mycobacterium tuberculosis, Brucella abortus, and Brucella suis*.


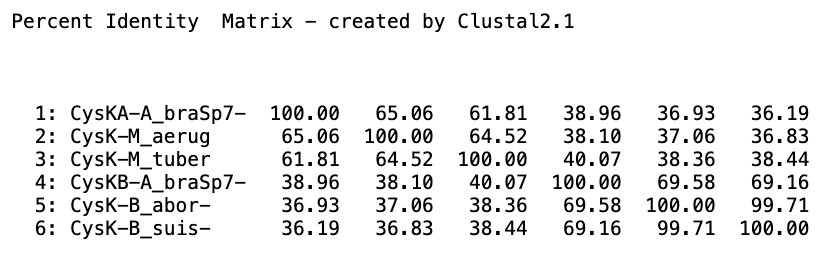


**Figure S14.** Percent identity matrix of protein alignment between CysK-A/B homologs generated by Clustal Omega. Strains: *A. brasilense* Sp7, *Microcystis aeruginosa*, *Mycobacterium tuberculosis*, *Brucella abortus*, *and Brucella suis*.
